# Characterizing Particulate Matter Impacts of Smoke From 2022 to 2023 Agricultural Burning in South Florida

**DOI:** 10.1029/2025GH001365

**Published:** 2026-01-27

**Authors:** Olivia Sablan, Bonne Ford, Emily Gargulinski, Giovanna L. Henery, Holly Nowell, Zoey Rosen, Kellin Slater, Amber J. Soja, Lisa K. Wiese, Christine L. Williams, Sheryl Magzamen, Emily V. Fischer, Jeffrey R. Pierce

**Affiliations:** ^1^ Department of Atmospheric Science Colorado State University Fort Collins CO USA; ^2^ Colorado State University Cooperative Institute for Research in the Atmosphere Fort Collins CO USA; ^3^ National Institute of Aerospace Hampton VA USA; ^4^ NASA Langley Research Center Hampton VA USA; ^5^ Department of Journalism and Media Communication Colorado State University Fort Collins CO USA; ^6^ Tall Timbers Tallahassee FL USA; ^7^ University of Oklahoma Institute for Public Policy Research and Analysis Norman OK USA; ^8^ Department of Environmental and Radiological Health Sciences Colorado State University Fort Collins CO USA; ^9^ Florida Atlantic University College of Nursing Boca Raton FL USA

**Keywords:** air quality, agricultural fire, smoke exposure, rural

## Abstract

Smoke from agricultural fires is a potentially important source of fine particulate matter (PM_2.5_) in the US. Sugarcane is burned in Florida to facilitate the harvesting process, with the majority of these fires occurring in the Everglades Agricultural Area (EAA), where there is only one regulatory air quality monitor. During the 2022–2023 sugarcane burning season (October–May), we used public low‐cost PurpleAir sensors, regulatory monitors, and 29 PurpleAir sensors deployed for this study to quantify PM_2.5_ from agricultural fires. We found satellite imagery is of limited use for detecting smoke from agricultural fires in Florida due to the cloud cover, overnight smoke, and the fires being small and short‐lived. For these reasons, surface measurements are critical for capturing increases in PM_2.5_ from smoke, and we used multiple smoke‐identification criteria. During the study period, median 24‐hour PM_2.5_ concentrations increased by 2.3–6.9 μg m^−3^ on smoke‐impacted days compared to unimpacted days, with smoke observed on 4%–28% of the campaign days (ranges from the different smoke‐identification criteria). Further, short‐term PM_2.5_ increases were observed over 40 μg m^−3^ during smoke events. We contrast the region near the EAA with large populations of low‐income and minoritized groups to the more affluent coastal region. The inland region experienced more smoke‐impacted monitor days than the Florida east coast region, and there was a higher study‐average smoke PM_2.5_ concentration in the inland area. These findings highlight the need to increase air quality monitoring near the EAA.

## Plain Language Summary

Agricultural fires are used in Florida to burn the leaves and tops of sugarcane plants for harvesting, mostly in the Everglades Agricultural Area (EAA). These fires produce smoke containing fine particulate matter (PM_2.5_), an air pollutant. Monitoring smoke from sugarcane fires is challenging in the EAA, as there is only one regulatory air quality monitor in the region. To study PM_2.5_ from agricultural‐fire smoke, we set up 29 low‐cost PM_2.5_ sensors (PurpleAir) in Florida from October 2022 to May 2023. Satellite observations were not helpful for detecting smoke in Florida because frequent cloud cover blocked satellites from observing the ground; satellites cannot capture nighttime smoke, and sugarcane fires are too short‐lived and small to detect. Median PM_2.5_ concentrations were higher on days with smoke, and smoke was present on 4%–28% of the days. Areas closer to the EAA had smoke more often and a higher study‐average smoke PM_2.5_ concentration compared to the coast. Higher percentages of low‐income and minoritized groups live close to the sugarcane region, making it important to monitor air quality in these communities. This study shows the importance of ground‐based monitors in places where satellites are limited and highlights the need for further research on agricultural fire smoke.

## Key Points

- Satellite observations of smoke and fires are of limited utility in southern Florida for detecting small, short‐lived agricultural burns
- During the 2022–2023 burning season, particulate matter (PM_2.5_) concentrations increased on days impacted by smoke in southern Florida
- The region closest to the agricultural area had more days impacted by smoke than the east coast of Florida, with higher total smoke exposure

## 1 Introduction

While wildfire smoke has been extensively studied, the impact of prescribed and agricultural smoke to air quality and human health remain less understood. Fine particulate matter (PM_2.5_) is a major air pollutant regulated by the US Environmental Protection Agency (EPA) under the National Ambient Air Quality Standards due to its adverse effects on human health (US EPA, 2024). A significant source of PM_2.5_ in the US is smoke from landscape fires, including agricultural burns, wildfires, and prescribed fires, which collectively accounted for 55% of total primary PM_2.5_ emissions nationally in 2020 and 57% in 2017 (US EPA, 2017, 2020). PM_2.5_ from wildfire smoke has been extensively studied and linked to increased risks of cardiorespiratory‐related emergency department visits (Alman et al., 2016; Gan et al., 2020; Hahn et al., 2021; Rappold et al., 2011) and hospitalizations (Gan et al., 2017; Magzamen et al., 2021; Stowell et al., 2019), adverse pregnancy outcomes (e.g., low birth weight) (Abdo et al., 2019; Holstius et al., 2012), and cognitive decline (Zhang et al., 2023). Research on the air quality impacts of agricultural and prescribed fires in the US has recently increased (Liu et al., 2016; Pennington et al., 2023; Sablan et al., 2024; Travis et al., 2023). Gaps in knowledge remain due to the challenges of monitoring these smaller, shorter‐duration burns, which often occur in rural areas with sparse regulatory and low‐cost monitoring networks.

Florida is the leading sugarcane producer in the US, with nearly half of the state's prescribed and agricultural burn authorizations being agricultural burns used to ease the sugarcane harvesting process (Baucum & Rice, 2009). Sugarcane burning involves the controlled burning of fields to remove the outer foliage of the plant, allowing easier access to cut the sugarcane stalks. Although there are environmentally friendly and health‐conscious methods of harvesting sugarcane (e.g., mechanical sugarcane harvesting), the use of fire is a cost‐effective and efficient method (Bordonal et al., 2018). Open Burned Authorizations must be obtained from the Florida Forest Service to burn sugarcane, and the authorizations show an average of about 50 acres burned per fire during 2020–2023. Fires are brief, typically lasting 15–20 min, but smoldering afterward can continue to produce smoke. The resulting smoke from sugarcane agricultural fires is an environmental and public health concern. Several recent studies have investigated sugarcane burning in the US (Nowell et al., 2022; Pinakana et al., 2023; Stem et al., 2024). By building on this foundational research, studies like ours aim to advance knowledge of sugarcane burning impacts on air quality.

In addition to exposure to smoke from agricultural sugarcane burning, southern Florida experiences smoke from prescribed fires, wildfires, and long‐range transported smoke from other regions (Brey et al., 2018). Prescribed fires are conducted on publicly managed land (e.g., national preserves, national and state parks, wildlife management areas) as well as private land, and often occur during the same period as agricultural fires. For example, during the 2022–2023 Florida sugarcane burning season, there were several wildfires that occurred in southern Florida, including the Mile 31 (National Centers for Environmental Information, n.d.‐a), Cypress Camp Trail (National Centers for Environmental Information, n.d.‐b), and Sandy wildfire (InciWeb, 2023). Additionally, long‐range transported smoke from landscape fire activity in other regions (e.g., Mexico, Cuba) can impact southern Florida. In this study, we remove long‐range transported smoke from our smoke estimates; however, prescribed fire smoke is not removed, as it occurs concurrently with sugarcane burning and is challenging to distinguish. This overlap of smoke from multiple fire types makes it challenging to isolate and quantify the contribution of agricultural smoke in Florida.

**Figure 1:**
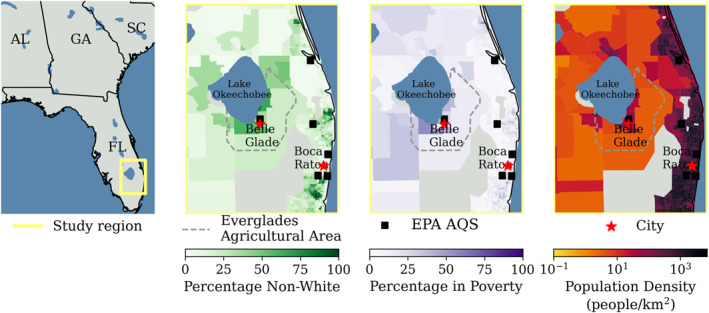
Demographics by census tract from the American Community Survey 2019 and EPA AQS PM_2.5_ monitor locations. (a) Map of southeastern US with the study region highlighted in a yellow box, (b) Percentage of people who identified as non‐white, (c) Percentage of people in poverty. Poverty is computed by the US Census Bureau using income and family size in conjunction with defined Poverty Thresholds. (d) Population density (people per km^2^) using a logarithmic scale. Gray areas have no reported population information. The Everglades Agricultural Area is outlined in a gray‐dashed line, and two major cities are identified with red stars.

Air pollution disproportionately affects low income and minoritized groups in the US (Chakraborty et al., 2022; Jbaily et al., 2022; Mohai et al., 2009). The Everglades Agricultural Area (EAA), where the majority of the sugarcane is grown in Florida, has higher percentages of people who identify as non‐White and people living in poverty compared to the coastal regions of south Florida (Figure 1). This area also has a lower population than the surrounding regions, with fewer regulatory monitors than the more populated coast. There was only one regulatory‐grade PM_2.5_ monitor in the EAA region during 2022–2023. We deployed PM_2.5_ monitors to investigate potential disparities.

This study aims to quantify the contribution of sugarcane burning to PM_2.5_ concentrations in southern Florida. By using a network of low‐cost PurpleAir sensors to supplement the region's regulatory monitors and existing public PurpleAir network, we measured PM_2.5_ during the 2022–2023 burning season. Our analysis works to isolate PM_2.5_ from agricultural‐fire smoke from other sources, including long‐range transported smoke and anthropogenic sources. Given the higher percentages of low‐income and minoritized populations living near the sugarcane growing region (Figure 1), our goal was to assess potential disparities in smoke exposure between the inland region, where sugarcane is grown, and the more affluent coastal areas. Previous work demonstrates that smoke from small landscape fires in this region can result in meaningful health impacts (Pennington et al., 2023). Our study seeks to fill in measurement gaps in assessing smoke exposure, which can provide better understanding of the health impacts.

## 2 Materials and Methods

**Figure 2:**
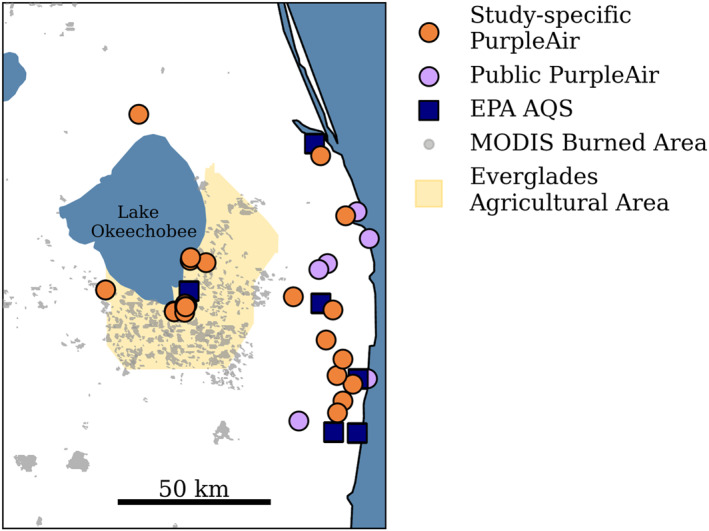
Locations of in situ PM_2.5_ monitors, including the PurpleAir deployed specifically for this study (orange), the Public PurpleAir (purple), and the EPA AQS (navy) with the Moderate Resolution Imaging Spectroradiometer (MODIS) burned area product. The Everglades Agricultural Area (EAA) is highlighted in yellow.

We deployed low‐cost PM_2.5_ sensors (PurpleAir) to southeastern Florida to determine the impact of smoke from sugarcane burning on PM_2.5_ concentrations for October 2022 – May 2023. We supplemented the regulatory monitors from the US Environmental Protection Agency Air Quality System (EPA AQS) (Figure 2). We used these in situ data alongside satellite observations from the National Oceanic and Atmospheric Administration Hazard Mapping System (NOAA HMS) (https://www.ospo.noaa.gov/products/land/hms.html) smoke plumes to attribute PM_2.5_ concentrations to smoke from burning. We used the Moderate Resolution Imaging Spectroradiometer (MODIS) burned area product (MCD64A1) and Burned Area Authorizations from the Florida Forest Service to determine the extent and timing of the sugarcane burning. We used the MODIS cloud fraction (MYD08_M3 v6.1; MOD08_M3 v6.1) for quantifying the cloud coverage in Florida to determine when fires or smoke may be missed by satellites. To investigate environmental conditions, we used the Modern‐Era Retrospective Analysis for Research and Applications, Version 2 (MERRA‐2) planetary boundary layer height reanalysis and the Automated Surface/Weather Observing Systems (ASOS/AWOS) wind speed data (https://www.weather.gov/asos/). We discuss these data in the following sections.

### 2.1 In Situ PM_2.5_ Measurements

We used in situ measurements from the EPA AQS and PurpleAir sensors in southeastern Florida to study PM_2.5_ emitted from sugarcane burning smoke. At the time of this study, there were six regulatory monitors in southeastern Florida within 50 km of the Everglades Agricultural Area (EAA) (Figure 2). Five of these monitors are stationed near the coast, approximately 25–50 km from burned areas (gray dots in Figure 2), where there is a higher population density. One regulatory monitor (AQS Site ID: 12‐099‐0008) was stationed in the city of Belle Glade, within the EAA. Before October 2021, this site was considered a non‐regulatory monitor, but has since been replaced with a regulatory monitor (Teledyne T640 at 5.0 LPM).

We supplemented the EPA AQS monitor by deploying 29 PurpleAir PM_2.5_ sensors. PurpleAir sensors are low‐cost devices (∼$300 USD) that use light scattering techniques to estimate PM_2.5_ mass (μg m^−3^). They include two Plantower sensors (channels A and B), which measure at 680 ± 10 nm. PurpleAir sensors also include a BOSCH BME280 to measure pressure, temperature, and humidity. They have been evaluated in the laboratory as well as in the field, and perform with high precision, but lower accuracy (Barkjohn et al., 2021; Jaffe et al., 2023; Malings et al., 2020; Tryner et al., 2020). Accuracy often decreases with increased temperature and humidity; however, we applied a correction factor to mitigate these issues (Barkjohn et al., 2021; full description in Section 2.1.1). The accuracy of PurpleAir measurements also decreases with increasing particle size. PurpleAir sensors perform best for particles between 0.3 and 1 μm, with limited ability to detect particles in the largest reported size range (2.5–10 μm) (Kuula et al., 2020; Molina Rueda et al., 2023). In this study, we aim to quantify smoke particles. Smoke that is measured is often aged (4–8 hr), and is typically around ∼0.3 μm (June et al., 2022), which is on the low end of the PurpleAir measurement range. Additionally, in regions with high ambient relative humidity, the hygroscopic growth of smoke particles can further increase their size and scattering properties, potentially causing inaccuracies in PurpleAir estimates.

#### 2.1.1 PurpleAir Field Deployment

PurpleAir sensors are commercially available and can be installed by the public. There were 6 public PurpleAir monitors in southeastern Florida at the time of this study (Figure 2). All 6 of these sensors were installed near the coast and outside of the EAA. To increase spatial coverage of PM_2.5_ monitoring in southern Florida, we partnered with volunteers throughout the region to host PurpleAir sensors (Figure 2). Due to the EAA being largely rural, we prioritized widespread placement. Previous studies have observed primary burning from October to March (H. Nowell et al., 2022); however, participants hosted monitors from September to June to capture the entirety of the burning season and gather non‐smoke PM_2.5_ concentrations outside of the burn season. During this period, the study region was affected by smoke from sources other than agricultural fires. This includes prescribed fire smoke, wildfire smoke, and smoke transported from other regions. As discussed below, we isolated the effects of smoke from agricultural sugarcane burning.

#### 2.1.2 PurpleAir Data Quality Procedures

PurpleAir calibrates the Plantower lasers in production; however, to use PurpleAir in a research context requires further corrections and validation. We tested the accuracy of the sensors in Fort Collins, CO from July to September 2022 against a Federal Equivalent Method (FEM) monitor (GRIMM EDM 180, Ainring, Germany). Sensors were co‐located (<5 m) with the GRIMM monitor for a period of 14 days. The 24‐hour average corrected PM_2.5_ concentrations during testing ranged from 2 to 29 μg m^−3^. The 24‐hour average temperature from the BOSCH BME280 ranged from 13 to 42°C, and the percent relative humidity ranged from 12% to 69%. We performed these tests to identify issues in individual PurpleAir sensors prior to deployment. We corrected the raw PM_2.5_ estimates with the Barkjohn et al. (2021) correction factor (discussed below). The average Pearson correlation coefficient during our testing between the two corrected PurpleAir channels within each individual sensor was 0.96, and the mean absolute difference was 0.59 μg m^−3^ (Figure S1 in Supporting Information S1). The correlation on average between the GRIMM monitor and the uncorrected PurpleAir measurements was 0.85 and the mean absolute difference was 3.62 μg m^−3^. After applying the correction factor to the PurpleAir estimates, the Pearson correlation on average was 0.64, and the mean absolute difference decreased to 2.03 μg m^−3^ (Figure S2 in Supporting Information S1). We identified one sensor to be unsuitable for deployment (*R* ^2^ = 0.03, mean absolute difference = 3.5, mean bias = −37.7%).

Due to an influx in available volunteers to host monitors in Florida, we also conducted brief quality testing prior to deployment with 6 sensors that were not included in the test above. We could not compare the PM_2.5_ estimates to the FEM during this testing period, so we compared to the average PM_2.5_ across all 6 sensors. We tested these monitors indoors for 1 day, before deeming them field‐ready. PM_2.5_ concentrations during this testing period were much lower than our previous testing in Fort Collins, CO. The 24‐hour indoor mean PM_2.5_ concentrations ranged from 0 to 1 μg m^−3^, with a standard deviation of 0.6 μg m^−3^. Due to the low concentrations and few observations with a brief testing period, the agreement between channels (*R* ^2^ = 0.2) and comparison to the overall sensor average (*R* ^2^ = 0.8) is relatively low (Figure S3 in Supporting Information S1); however, the sensors performed with consistently high precision with a maximum difference of channel agreement of 0.13 μg m^−3^.

Before analysis of the campaign data, we performed several quality checks of the raw PurpleAir data (“CF1”) using the methods outlined in a previous study (Sablan et al., 2024). We took 10‐min averages of the PM_2.5_ estimates and then removed data with the following conditions: (a) temperature >65°C (0.0092% of observations), (b) relative humidity >100% (0.0007% of observations), (c) channel disagreement >10% from the average of the two channels or 10 μg m^−3^ in the absolute difference between the channels (1.4% of observations), and (d) measurements >500 μg m^−3^ (0.0007% of observations).

We applied the Barkjohn et al. (2021) correction factor to all the quality‐checked 10‐min average PurpleAir data from the deployment in Florida. This correction factor was developed for the entire US and scales PM_2.5_ based on measurement concentrations and relative humidity. The US EPA developed the correction factor using measurements from 53 PurpleAir sensors co‐located with regulatory monitors across 16 states, including one site in Florida. At the co‐located site in Florida, the average relative humidity (60%) was similar to this campaign (56%). Across all monitors in the study, the correction factor improved the root mean square error of the PurpleAir compared to the reference monitors by >60%.

Additionally, we tested two other PurpleAir correction factors available on the PurpleAir website (https://map.purpleair.com/), the ALT [cf = 3] correction factor (Wallace et al., 2021), and the AQandU correction factor (Kelly et al., 2017; Sayahi et al., 2019). We compared the average PM_2.5_ concentrations from the 14 PurpleAir sensors in Belle Glade and the EPA regulatory monitor (AQS Site ID: 12‐099‐0008) for the raw PurpleAir and the three correction factors. The Barkjohn et al. (2021) correction factor yielded PM_2.5_ estimates closer to the EPA reported values for this field campaign (higher *R* ^2^; lower mean absolute difference) than other correction factors (Figure S4 in Supporting Information S1). While this comparison is limited by the lack of direct co‐location between the monitors, it provides some indication of which correction factor may be most appropriate for this study.

All measurements were converted from UTC to Eastern Time (ET). We accounted for daylight savings during this campaign, and PM_2.5_ concentrations were shifted an hour later after 12 March 2023.

### 2.2 Administrative Burn Permitting Data

We investigated the timing of sugarcane burning in Florida by obtaining Open Burn Authorizations (OBAs) from the Florida Fire Service (Florida Department of Agriculture and Consumer Services, 2025). An OBA is required for agricultural burning, silvicultural burning (burning for land management), land clearing, pile burning, and acreage burning (Florida Department of Agriculture and Consumer Services, 2024). We received statewide OBAs for October 2020–October 2023. Sugarcane agricultural burns accounted for 45% of all issued OBAs during this period, and most of the sugarcane burned areas are within or slightly west of the Everglades Agricultural Area (Figure S5 in Supporting Information S1). Sugarcane burns were ignited starting at 06:00 and continued until 16:00 ET (Figure S6 in Supporting Information S1); however, Florida regulations require most fires to be ignited at 09:00 ET or later. The burns must be completed 2 hrs after sunset. There were other types of fires that occurred during the campaign, including several ecological prescribed fires conducted in February 2023 near the EAA (Figure S7 in Supporting Information S1).

### 2.3 Satellite Products

We used the MODIS burned‐area product to determine the extent of sugarcane burning. To designate smoke impacts, we used the NOAA HMS smoke plumes and fire hotspots. Satellite products can be useful for distinguishing the presence of smoke, although we found several drawbacks with using NOAA HMS in this region to study small agricultural fires (discussed further in Sections 2.3.3 and 2.4). We used the MODIS cloud‐fraction products from Aqua and Terra for investigating the potential for clouds to obscure satellite detection of smoke. Additionally, we used the MERRA‐2 planetary boundary layer height reanalysis data and ASOS wind speed to understand smoke dispersion due to environmental factors in the region of study.

#### 2.3.1 Burned Area Product

We used the MODIS/Terra + Aqua Direct Broadcast Burned Area Monthly L3 Global 500 m SIN Grid V061 product (Giglio et al., 2021) to determine the extent of burning in southeastern Florida (Figure 2). The MODIS burned area product has been shown to underpredict burned area (Chang & Song, 2009; Mohler & Goodin, 2012; Scholtz et al., 2020; Zhu et al., 2017); therefore, we did not use this product as a method of quantification of the impact of agricultural fires, but instead used it to qualitatively highlight the region of burning.

#### 2.3.2 Cloud Fraction and Wind Data

To investigate the presence of clouds in Florida, we used the MODIS/Aqua Aerosol Cloud Water Vapor Ozone Monthly L3 Global 1Deg CMG (MYD08_M3) and the MODIS/Terra Aerosol Cloud Water Vapor Ozone Monthly L3 Global 1Deg CMG (MOD08_M3) data (Platnick, 2015). We took an area average of this product for our study region (−81° ≤ x ≤ −80°W; 25° ≤ x ≤ 27°N) for each month to investigate seasonal variability. We conducted the same area averaging using planetary boundary layer height (PBLH) reanalysis from MERRA‐2 Hourly 0.625 × 0.5° V5.12.4 (M2T1NXFLX) data (Global Modeling and Assimilation Office (GMAO), 2015). We obtained wind speed observations from the ASOS station in Clewiston, FL (−80.95°W, 26.75°N). Although this station is not within the EAA, it is closest ASOS to the burning region in Florida. We used PBLH and wind speeds for investigating smoke dispersion.

#### 2.3.3 NOAA HMS Smoke and Fire Products

We used smoke plumes and fire hotspots from the NOAA HMS Fire and Smoke Product (Rolph et al., 2009; Ruminski et al., 2023). NOAA HMS uses satellite observations from geostationary and polar‐orbiting satellite observations. Digitization of smoke plumes as polygons and automation of fire hotspot locations are quality checked by analysts. By taking into account multiple satellite observations, the HMS is a comprehensive data set of smoke plumes and fire hotspots; however, relying on this product entirely may not be adequate to distinguish the presence of smoke in certain regions, as discussed in the next section. There are several limitations with the HMS product. HMS smoke polygons are only produced during the daytime. Satellite observations cannot be used overnight to digitize smoke plumes due to the lack of visible imagery. In regions where there is overnight smoke, there is no information provided regarding nighttime plumes. Additionally, when there is dense cloud cover during the day, smoke may be hidden or obscured and missed by HMS analysts. The source location of the smoke plume is not identified in the HMS data set.

### 2.4 Smoke Designation via Multiple Methods

Previous studies have used HMS smoke plume polygons to determine the presence of smoke either in the atmospheric column or at the ground (e.g., Brey & Fischer, 2016; Corwin et al., 2022; O’Dell et al., 2019; Sablan et al., 2024). However, there are several limitations of this product that are particularly important for agricultural burning in the study region. While HMS incorporates data from polar‐orbiting and geostationary satellites, smaller fires often go undetected. Despite HMS using a suite of satellite observations, both polar‐orbiting and geostationary satellites are not able to detect smaller fires: the two overpass times of polar‐orbiting are not when burns typically occur (Al‐Saadi et al., 2008; Huang et al., 2018; Nowell et al., 2018; Soja et al., 2009).

Additional challenges in this region include the daytime‐only availability of HMS smoke polygons and frequent cloud cover that obscures satellite observations. The HMS product is limited to daytime observations, yet we observed smoke overnight in the EAA region, indicated by large increases in PM_2.5_ concentrations (Figure S8 in Supporting Information S1; Figure 5). Cloud cover further inhibits satellite‐based detection; during the burning season (October 2022 – May 2023), the mean cloud fraction from MODIS products was 0.5 over southeastern Florida (Figure S9 in Supporting Information S1). The highest monthly values were in April and ranged from 0.60 to 0.67, depending on the satellite and time of day. We provide results from using HMS smoke polygons alone to designate smoke; however, these values should be used as a comparison to other methods, and we do not suggest relying only on HMS smoke polygons alone to designate smoke in this region because of the issues described in this section.

The HMS product is, however, well‐suited to identify the presence of large smoke plumes from fires upwind. Transported smoke from fires outside the study regions (e.g., Mexico, Cuba) reached Florida during our campaign. We used the size of the HMS smoke plumes to separate transported smoke from local‐fire smoke. When smoke plumes had a total area >15° squared, we classified periods as impacted by smoke transported to Florida from other regions. This area threshold was determined by an analysis of historical HMS smoke plume areas in Florida.

Given the particular challenges associated with this region that is impacted by local agricultural burning at various times of day and frequently under cloud conditions, we conservatively designated the presence of smoke on a daily basis using multiple methods:

1. 15 μg m^−3^: A day at a specific monitor is designated as smoke‐impacted when PM_2.5_ concentrations ≥15 μg m^−3^ for at least 1 hour within that 24‐hour period.
2. 20 μg m^−3^: A day at a specific monitor is designated as smoke‐impacted when PM_2.5_ concentrations ≥20 μg m^−3^ for at least 1 hour within that 24‐hour period.
3. 30 μg m^−3^: A day at a specific monitor is designated as smoke‐impacted when PM_2.5_ concentrations ≥30 μg m^−3^ for at least 1 hour within that 24‐hour period.
4. 20 μg m^−3^ or HMS: A day at a specific monitor is designated as smoke‐impacted when either PM_2.5_ concentrations are ≥20 μg m^−3^ threshold for at least 1 hour within that 24‐hour period or a NOAA HMS smoke plume polygon overlaps the monitor. A flowchart describing this method can be found in Figure S10 of Supporting Information S1.

Our analysis uses several PM_2.5_ thresholds (1: 15 μg m^−3^, 2: 20 μg m^−3^, 3: 30 μg m^−3^) to provide an estimated range of the impact of local agricultural burning on 24‐hour average PM_2.5_. We compared the number of monitor days identified by these ground‐based thresholds to those identified using only the presence of HMS smoke plumes (Table S1 in Supporting Information S1). Although many monitor days had an HMS smoke plume overhead but did not meet our thresholds, HMS plumes alone do not capture all smoke events; therefore, our threshold designation methods are necessary. The multi‐threshold approach is important because without reliable satellite‐based methods, misclassification of PM_2.5_ as smoke is possible, and we need to estimate the uncertainty in smoke impacts. In particular, there may be other sources of PM_2.5_, particularly in the coastal region, that may cause PM_2.5_ concentrations to exceed these thresholds. We provide results both with and without the removal of transported smoke in Table 1, but focus the following discussion on local smoke impacts, with transported smoke excluded.

We also used a combined technique using HMS smoke plumes and an hourly PM_2.5_ threshold of 20 μg m^−3^. We acknowledge that 20 μg m^−3^ may not be an absolute threshold for agricultural smoke in Florida, but in this study, the presence of smoke often pushed PM_2.5_ concentrations above this threshold for one to several hours. This is further supported by previous studies (e.g., Nowell et al., 2022), which show that PM_2.5_ concentrations in this region remain well below 20 μg m^−3^ in the absence of smoke. A monitor was designated to be smoke‐impacted for the day when there was an HMS smoke plume over the monitor or when there was a one hourly average during 24‐hour that reached or exceeded 20 μg m^−3^. A monitor was designated to be smoke‐free for the day when both of the following are true: (a) no HMS smoke plumes were observed over the monitor; and (b) all hourly average PM_2.5_ concentrations remained <20 μg m^−3^.

Given the relatively small size of smoke plumes from agricultural fires in Florida, we categorized plumes as either from local fires or transported from upwind fires based on their area. Periods with transported smoke in the atmospheric column may coincide with local burning, further increasing surface PM_2.5_ concentrations. However, transported smoke does not always reach the surface. To account for this, we provide estimates of the impact of local agricultural smoke both with and without the removal of days potentially influenced by transported smoke. Due to the challenges with HMS plumes over Florida, the number of days impacted by transported smoke is likely a lower bound. The threshold designation methods used may lead to a misclassification of PM_2.5_ as smoke with lower thresholds might include contributions from other sources. In contrast, the higher threshold may miss lower‐concentration smoke events, leading to an underestimation of smoke and its impacts. Additionally, the use of a higher threshold could overestimate median smoke concentrations on smoke‐impacted days for the high threshold designation, while underestimating the lower threshold. However, the upper threshold may be an underestimate of the annual and seasonal contribution of smoke to PM_2.5_ because there are fewer days classified as smoke‐impacted.

### 2.5 Trajectory Analysis to Support Tracking of Smoke Transport

To track the near‐source transport of smoke from fires in Florida, we used the NOAA HYbrid Single‐Particle Lagrangian Integrated Trajectory (HYSPLIT) model (Stein et al., 2015). This model is commonly used to compute the trajectory of air parcels. We calculated 12‐hour forward trajectories initiated from all HMS fire hotspots in southern Florida (−81.5° × −80°; 26° × 27.5°) during the campaign (October 2022 – September 2023). The HMS fire hotspot product combines detections from the Geostationary Operational Environmental Satellite—*R* Series (GOES‐R)/Advanced Baseline Imager (ABI) Fire Detection product and the Joint Polar Satellite System (JPSS)/Visible Infrared Imaging Radiometer Suite (VIIRS) products, and NASA's Earth Observing System (EOS) Moderate Resolution Imaging Spectroradiometer (MODIS) fire product. We used meteorological data from the NOAA High‐Resolution Rapid Refresh (HRRR) model (Dowell et al., 2022), which provides conditions every 4 hr. The HYSPLIT model produces trajectory locations for every hour; however, we interpolated between the reported locations to provide 10‐min observations. This finer temporal resolution allows for tracing of short‐lived plumes and more accurate determination of smoke transport, which may vary significantly on a sub‐hourly basis due to shifting wind patterns and rapid changes in plume behavior. If a calculated trajectory intercepts the surface (*z* = 0), all subsequent locations for this trajectory were removed (Stein et al., 2015). We acknowledge regional differences in weather patterns (e.g., clouds) may make it more challenging to detect hotspots in certain areas, possibly leading to a bias in the HYSPLIT results as winds may, on average, be different under clear sky versus cloudy conditions.

## 3 Results

### 3.1 Duration of Burn Season in Southern Florida

**Figure 3:**
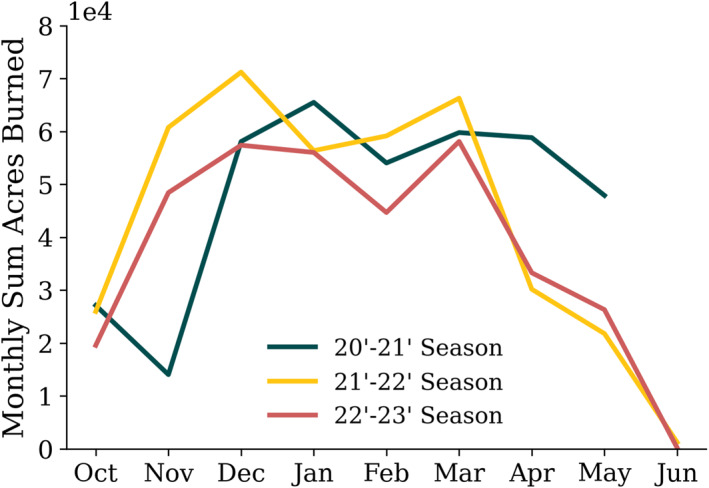
Monthly acres burned from sugarcane‐agricultural fires from the Florida Fire Service Open Burned Authorizations (OBAs) for 2020, 2021, 2022, and 2023, separated into burning seasons (October – June). There were no OBAs issued for July–September. There were 15 sugarcane agricultural fires away from the study region (>27.5°N) that were not included in this analysis.

To determine the length of the current burning season in Florida, we obtained Open Burn Authorizations (OBAs) for sugarcane burning near the study region (≤27.5°N) from the Florida Fire Service (Figure 3). In recent years, the burning season has extended from October through May. Previous studies (and reports) also indicate the sugarcane burn season occurs in Florida from October to May (Baucum & Rice, 2009; McCarty, 2011; Nowell et al., 2022; Sugar Field Burning,” 2024). The number of acres burned during the season of study (2022–2023) was consistent with the previous two seasons. The majority of the burning during the three seasons occurred from December through March, with a monthly maximum of 71,200 total acres burned during December 2021. There is a seasonal average of 374,100 total acres burned. These seasonal averages are in line with Bacuum & Rice et al. (2009) who found a decrease in sugarcane acreage burned over time, with 454,400 acres during the 2000–2001 season and 400,000 acres during the 2008–2009 season. There is interannual variability in the total burning each season; 393,000 acres were burned in 2021–2022 while only 344,000 acres were burned over the course of the 2022–2023 burning season.

### 3.2 Impact of Smoke on PM_2.5_ Concentrations

**Figure 4:**
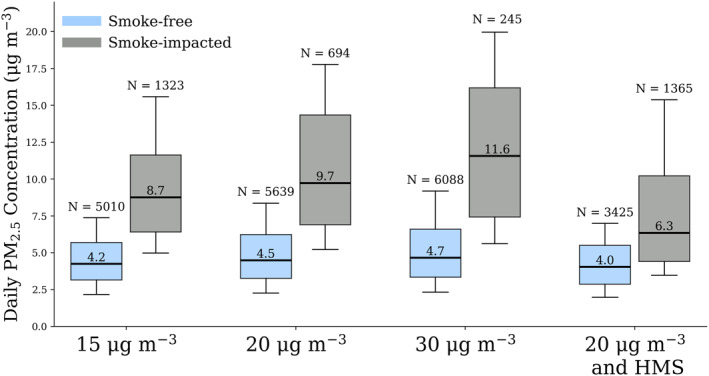
24‐hour average PM_2.5_ concentration distributions for different smoke designation methods during the 2022–2023 burning season for all sensors in southern Florida. Outliers have been excluded from the figure and the median for each category is shown above the bold line. The edges of the boxes represent the 1st and 3rd quartiles. The whiskers are 10th and 90th percentiles. Days impacted by transported smoke were not removed from the distributions.

In this section, estimates of the impact of smoke on PM_2.5_ are presented by number of days per monitor or “monitoring days” (Figure 4; Table 1). In Table 1, each designation method is compared (see Section 2.4). The median 24‐hour PM_2.5_ concentrations on smoke‐free days ranged from 4.0 to 4.7 μg m^−3^ and from 6.3 to 11.6 μg m^−3^ for smoke‐impacted days, and the percent of monitoring days deemed as smoke‐impacted ranged from 4% to 28% (Figure 4). The median contribution of local agricultural fires to PM_2.5_ ranged from 2.3 to 6.9 μg m^−3^ across all designation methods, with the inclusion of potential transported‐smoke days. With the transported smoke removed, this range decreases to 1.4–5.1 μg m^−3^. Transported smoke‐days may also be impacted by local smoke; therefore, removing transported smoke days may also remove some local‐smoke influence. The seasonal contribution of smoke to PM_2.5_ was calculated by multiplying the median smoke contribution to PM_2.5_ by the fraction of days affected by smoke for each monitor during the burning season (October – May), then dividing by the total number of monitor observation days during the season. The seasonal contribution ranges from 0.27 to 0.94 μg m^−3^, including days potentially impacted by transported smoke. Under the assumption that agricultural burning is entirely during October – May (Figure 3), the median contribution of smoke to the 2022–2023 annual‐average PM_2.5_ was 0.13–0.43 μg m^−3^, including transported‐smoke days. If days potentially influenced by transported smoke are excluded, the annual contribution ranged from 0.06 to 0.16 μg m^−3^.

**Table 1:** Number of Smoke‐Free and Smoke‐Impacted Days at Each Monitoring Site, the PM_2.5_ Concentration (Median (25th–75th)) for Smoke‐Free and Smoke‐Impacted Days, the Smoke Contribution to PM_2.5_, the Seasonal and Annual Contributions of Smoke PM_2.5_ for Each Smoke Designation Method in μg m^−3^

Smoke designation method	Number of smoke‐ free monitoring days	Number of smoke‐ impacted monitoring days	Concentration (μg m^−3^) on smoke‐ free days median (25th–75th)	Concentration (μg m^−3^) on smoke‐ impacted days median (25th–75th)	Smoke contribution to PM_2.5_ on smoke‐impacted days (μg m^−3^)	Seasonal contribution of smoke to PM_2.5_ (μg m^−3^)	Annual contribution of smoke to PM_2.5_ (μg m^−3^)
>15 μg m^−3^	5,010	1,323	4.2 (3.1–5.7)	8.7 (6.4–11.6)	4.5	0.94	0.43
	3,745	743	4.0 (2.9–5.2)	7.0 (5.6–9.6)	3.1	0.51	0.16
>20 μg m^−3^	5,639	694	4.5 (3.3–6.2)	9.7 (6.9–14.3)	5.2	0.57	0.26
	4,096	392	4.1 (3.0–5.5)	7.8 (5.9–11.4)	3.6	0.32	0.10
>30 μg m^−3^	6,088	245	4.7 (3.3–6.6)	11.6 (7.4–16.2)	6.9	0.27	0.13
	4,340	148	4.3 (3.1–5.8)	9.4 (6.4–13.6)	5.1	0.17	0.06
>20 μg m^−3^ and HMS	3,425	1,365	4.0 (2.9–5.5)	6.3 (4.4–10.2)	2.3	0.66	0.23
	3,425	1,063	4.0 (2.9–5.5)	5.4 (4.1–7.6)	1.4	0.32	0.10
Designation included for completeness, but not the best estimate due to HMS limitations in the study region:							
HMS only	3,689	2,644	4.2 (3.0–5.8)	5.9 (4.2–8.8)	1.7	0.70	0.33
	3,689	799	4.2 (3.0–5.8)	4.8 (3.9–6.7)	0.6	0.11	0.04

*Note.* Designations with removal of transported smoke (determined from the NOAA Hazard Mapping System) smoke plumes are in gray rows. The “HMS only” smoke designations are included for completeness, but they are not the best estimate due to HMS limitations in the study region.

Nowell et al. (2022) showed that sugarcane harvest activities contributed 1.4 μg m^−3^ to seasonal mean PM_2.5_ from 2009 to 2018. They subtracted the mean PM_2.5_ outside the harvesting season (April – September) from PM_2.5_ concentrations measured at the regulatory site in Belle Glade during the harvest season (October – March). The upper end of our seasonal contribution to smoke was 0.94 μg m^−3^. Differences in these estimates are likely due to variations in the smoke season length, different calculation methods, and potential seasonal cycles in non‐smoke PM_2.5_, which may have influenced the results. Despite inherent differences in methodology, the results are consistent and the growing bodies of research show that PM_2.5_ concentrations increase with burning in this region of Florida.

### 3.3 Variability in the Diurnal Cycle of PM_2.5_

**Figure 5:**
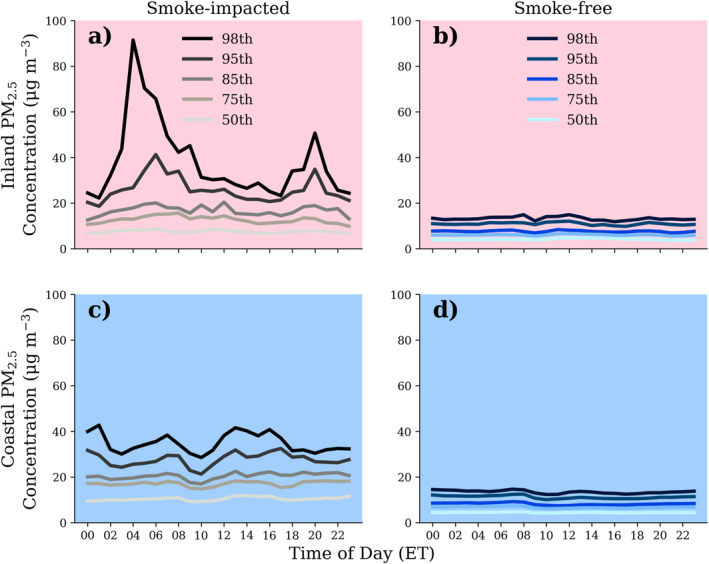
Hourly 50th, 75th, 85th, 95th, and 98th percentile PM_2.5_ concentrations during October 2022 – May 2023 for (a) inland monitors (>50 km from the coastline; pink) on smoke‐impacted days (classified using criteria of an hourly average PM_2.5_ concentration ≥20 μg m^−3^), (b) inland monitor on smoke‐free days, (c) coastal monitors (<30 km from coastline; blue) on smoke‐impacted days, and (d) inland monitors on smoke‐free days. Other smoke designation methods are shown in Figures S11–S13 of Supporting Information S1.

Diurnal patterns of smoke PM_2.5_ concentrations in the region were evaluated using the ≥20 μg m^−3^ threshold (Figure 5). Results for all other designation methods are provided in the Figures S10–S12 of Supporting Information S1. PM_2.5_ increases related to agricultural burning occurred at varying times throughout the day (e.g., early morning, mid‐morning, afternoon, evening, and overnight). As a result, when averaging across all hours for the entire campaign, there is not a distinct diurnal cycle.

On days designated as smoke‐impacted, there is an increase in the hourly average 98th percentile of PM_2.5_ at inland sites overnight on smoke‐impacted days that does not occur on smoke‐free days. This pattern is not evident at the coastal monitoring locations. Two inland local maxima in PM_2.5_ occurred at 04:00 (91 μg m^−3^) and 20:00 ET (51 μg m^−3^; Figure 5). This particular timing may be a result of (a) fires smoldering overnight, (b) lower planetary boundary layer heights (PBLH) decreasing dispersion and transport of smoke away from local sources, and (c) lower wind speeds at night decreasing ventilation. Although authorized burns are generally limited to daytime hours (09:00 ET to 2 hrs before sunset), residual smoldering can produce smoke well into the night, when stable atmospheric conditions can concentrate smoke near the surface. The PBLH in the study region during the burn season (October 2022 – May 2023) averaged 574.7 m from 23:00 to 07:00 EST. The PBLH increased to 1,369.9 m at 14:00 ET. Average wind speeds in this region are also lower overnight; the average wind speed from 23:00 to 07:00 ET was 4.2 km hr^−1^, with a maximum wind speed of 7.0 km hr^−1^ at 14:00 ET.

Nowell et al. (2022) reported that seasonal‐average PM_2.5_ concentrations from the Belle Glade regulatory monitor maximized at 10:00 ET during October–March 2009–2019, and decreased after 19:00 ET. Note that this averaging included days with local burning and without. This pattern of elevated PM_2.5_ concentrations in the late morning was observed both during the October–March harvest season and outside of it. In our study, smoke was observed at varying times throughout the day, with inland PM_2.5_ increases overnight and in the early morning due to meteorological conditions and/or smoldering. These increases on smoke‐impacted days were most evident in the 98th percentile of hourly averages, but there were also small increases in median PM_2.5_ concentrations at 06:00 and 20:00 ET. Together, both studies are important in distinguishing PM_2.5_ increases from smoke in this region.

### 3.4 Determining Regional Impacts of Smoke PM_2.5_

**Figure 6:**
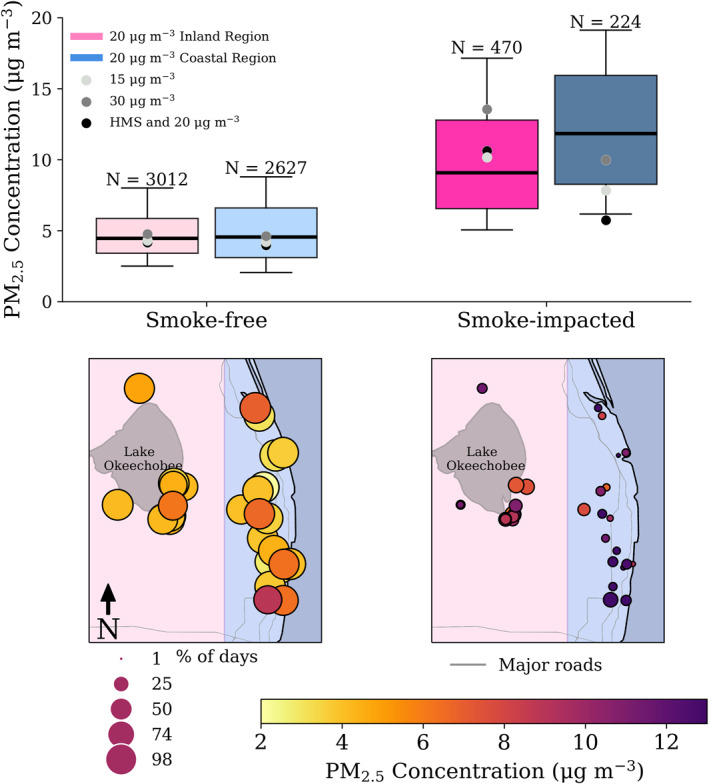
Top Row: Boxplots of daily average PM_2.5_ for smoke‐free days and smoke‐impacted days using the ≥20 μg m^−3^ designation method. The distributions are separated by monitors near the coast (blues) and monitors in the inland region (pinks). Outliers have been excluded and the bold line represents the median for each category. The edges of the boxes represent the 1st and 3rd quartiles. The whiskers are 10th and 90th percentiles. Bottom Row: Maps of median PM_2.5_ concentrations at each monitor on smoke‐free (left) and smoke‐impacted days (right). Markers are sized by the corresponding percentage of days that meet each criteria (smoke‐impacted or smoke‐free). Map regions are shaded by the inland versus coastal designation. Note: the color bar has a lower limit of 2 μg m^−3^. Other smoke designation methods are shown in Figures S13–S15 of Supporting Information S1.

Using the ≥20 μg m^−3^ threshold for designating smoke, monitors in the inland region were impacted by smoke approximately twice as often as monitors in the coastal region during the 2022–2023 burning season (Figure 6). Specifically, 13% of inland monitor days were deemed smoke‐impacted compared to 8% of days for coastal monitors. However, when the coastal region was impacted by smoke, the median observed daily PM_2.5_ concentration was 11.8 μg m^−3^ compared to 9.1 μg m^−3^ for the inland region. On smoke‐free days, concentrations were similar between regions, with a median PM_2.5_ concentration of 4.5 μg m^−3^ for the coastal monitors and 4.4 μg m^−3^ for the inland monitors. While there were fewer instances of smoke‐impact at the coastal monitors, the smoke‐impacted periods were characterized by slightly higher median PM_2.5_ concentrations.

Extreme smoke events (PM_2.5_ > 40 μg m^−3^) during the 2022–2023 agricultural burning season also show distinct temporal and spatial patterns (Figure S17 in Supporting Information S1). Inland monitors had more frequent periods of PM_2.5_ > 40 μg m^−3^ during early morning and evening hours compared to the coastal region. This suggests that smoke may persist overnight, potentially due to nighttime smoldering or atmospheric conditions that limit dispersion and ventilation. These spatial differences further support the hypothesis that smoke exposure differs regionally, with inland areas experiencing more extreme smoke exposure compared to the coastal region.

**Figure 7:**
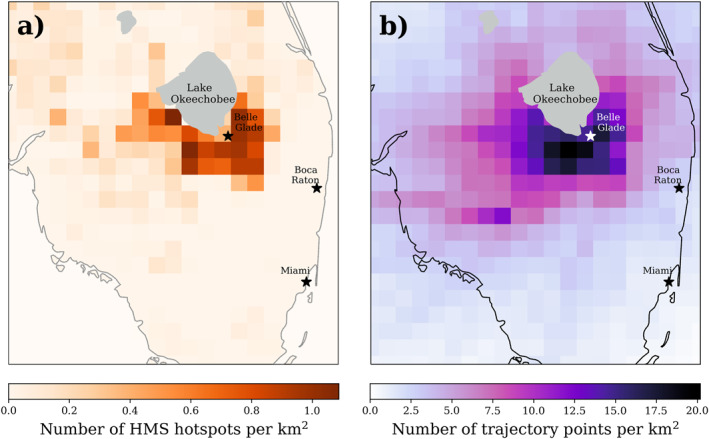
(a) Sum of gridded HMS fire hotspots per gridded area for the October–May 2022–23, (b) Sum of 10‐min interpolated 12‐hour forward trajectories per gridded area initiated from each HMS fire hotspot from the NOAA HYSPLIT model.

The NOAA HYSPLIT model was used to further explore the regional differences of smoke transported from agricultural burning (Figure 7). We calculated 12‐hour forward trajectories initiated from HMS fire hotspots, which includes detections from the GOES‐R/ABI Fire Detection product, the JPSS/VIIRS products, and the EOS MODIS product. The maximum number of HMS hotspots (1.1 hotspots per km^2^) was detected south‐southwest of Lake Okeechobee, coinciding with the region of maximum HYSPLIT trajectory points south of the lake (19.4 points per km^2^). Due to the limitations with HMS in this region, this analysis may provide an estimate of the minimum difference in smoke exposure between regions. When considering all trajectories on days where HMS hotspots were detected, 26% of the total trajectories moved westward (≤−81.36°W), 21% moved eastward (≥−80.36°W), and the majority of trajectories (49%) stayed within the bounds (−81.2° ≤ x ≤ −80.36°W) in the agricultural region (Figure S18 in Supporting Information S1). If we only consider trajectories that transport away from the agricultural region, 59% of trajectories moved westward (≤−81.36°W) and 41% moved eastward (≥−80.36°W). These findings are consistent with Figure 6, as there were fewer smoke‐impacted days during the campaign for the coastal monitors (224 total days) than the inland monitors (470 total days).

## 4 Conclusions and Limitations

Air quality in rural regions is often under‐monitored. To fill monitor gaps in the rural Everglades Agricultural Area (EAA) in Florida, citizen scientists hosted 29 PurpleAir sensors. To quantify smoke from sugarcane burning on PM_2.5_ concentrations from October 2022 to May 2023, we incorporated data from study‐specific and public PurpleAir, as well as regulatory monitors. Due to the limitations of satellite observations in the study region and the agricultural fires being short‐lived and small, several smoke designation methods were used to quantify the uncertainties in smoke PM_2.5_ estimates. These methods include using three thresholds of PM_2.5_ (15 μg m^−3^; 20 μg m^−3^; 30 μg m^−3^) and a method that uses a combination of HMS observations and the 20 μg m^−3^ threshold. Median 24‐hour PM_2.5_ concentrations increased by 2.3–6.9 μg m^−3^ on smoke‐impacted days compared to unimpacted days, with 4%–28% of days between October 2022 and May 2023 exhibiting smoke influence.

Smoke was observed to occur throughout all hours of the day near the burning region, although most of the agricultural‐fire smoke occurred during the daytime. Due to atmospheric conditions and the potential for overnight smoldering, there were cases of smoke during the evening and early morning hours. A distinct, consistent diurnal cycle in median smoke PM_2.5_ for inland or coastal monitors was not identified; however, there were extreme smoke events (e.g., 98th percentile; hours >40 μg m^−3^) overnight.

During this study, the inland region, near the EAA, experienced more smoke‐impacted days than the coastal region. The median PM_2.5_ on smoke‐impacted days in the coastal region was 11.8 μg m^−3^ compared to 9.1 μg m^−3^ for the inland region. This result suggests that smoke transport to the coast is infrequent but results in higher PM_2.5_ concentrations when it occurs. The HYSPLIT model was used to calculate the trajectories of air parcels initiated from identified HMS hotspots to understand the predominant transport pathways of smoke from agricultural fires in this region. The majority of trajectories moved westward, away from the east coast, consistent with less frequent smoke impacts in the coastal region. The greater frequency of smoke impact in low‐income, rural, inland regions emphasizes the critical need to expand air quality monitoring to these underserved areas (US Census Bureau, 2023).

Overall, satellite products were not well suited for identifying smoke and fires in this region due to: (a) the consistent presence of clouds, (b) overnight smoke, and (c) the occurrence of many small, short‐lived fires, which satellites are not able to accurately quantify. Products that rely on HMS to identify smoke (e.g., O’Dell et al., 2019) to diagnose the presence of smoke will likely underestimate smoke at the surface in southern Florida, particularly from local fires. Without consistent satellite observations, resources to detect the presence of smoke are limited. Alternative approaches, such as using surface‐based measurements, can be used to identify the presence of smoke. In this study, multiple methods were used to designate smoke‐impact that leveraged the surface measurements. Given the strengths and weaknesses of our methods, the estimations in this manuscript provide a lower and upper bound of smoke exposure during the study.

Isolating the impacts of sugarcane agricultural fires from other fire types presents inherent challenges. While this analysis focused on the primary sugarcane burning season (October–May), other fires also occurred during this period, including prescribed (silvicultural) burns for ecological purposes, agricultural fires for pasture and range management, and wildfires. These overlapping fire activities may have contributed to overestimating the smoke PM_2.5_ attributed to sugarcane burning.

The use of low‐cost PurpleAir sensors may introduce uncertainty to PM_2.5_ measurements. PurpleAir sensors have been shown to measure a low bias at low concentrations and high bias at high concentrations (Sayahi et al., 2019; Tryner et al., 2020). Most measurements in this study were at low concentrations due to the infrequency of smoke‐impacted days, likely leading to an underestimation of PM_2.5_. To address this, we applied the Barkjohn et al. (2021) correction factor. Despite inherent biases, PurpleAir sensors provide valuable data in regions with limited monitoring resources.

This study contributes to the growing understanding of smoke impacts from agricultural sugarcane burning in Florida. While the results highlight differences in smoke exposure between inland and coastal areas, they also show the need for improved monitoring strategies to capture fine‐scale and short‐lived smoke events. Satellite‐based approaches for smoke identification face challenges in this region due to frequent cloud cover, the small size of fires, and the presence of overnight smoke. Nighttime smoke exposure is particularly concerning, as residents in nearby agricultural areas, many of whom are low‐income and lack air conditioning, may unknowingly leave their windows open to cool their homes. This behavior can exacerbate health risks from smoke exposure. It is essential to implement measures to reduce smoke exposure. We recommend strategies such as encouraging residents to keep windows closed at night and use air conditioning, air purifiers, or box fans with filters to improve indoor air quality. Future research should further explore the health impacts of sugarcane agricultural fire smoke exposure, a critical yet understudied area in this region, and accurate smoke exposure estimates are fundamental for determining health impacts.

## Conflict of Interest

The authors declare no conflicts of interest relevant to this study.

## Supporting information

Supporting Information S1

## Data Availability

The PurpleAir data from the field deployment can be downloaded from Sablan et al. (2025). The PurpleAir data from public monitors can be accessed through the PurpleAir API, and the details can be found here: https://community.purpleair.com/t/making‐api‐calls‐with‐the‐purpleair‐api/180. The EPA regulatory PM_2.5_ data is publicly available and can be downloaded from https://aqs.epa.gov/aqsweb/airdata/download_files.html. The MODIS MCD64A1 burned area product is from Giglio et al. (2021). The NOAA/NEDIS Hazard Mapping System Smoke Product is available at “Hazard Mapping System Smoke Product,” (2022) and the Fire Product is available at “Hazard Mapping System Fire Product,” (2022). Administrative Open Burn Authorizations from the Florida Fire Service can be requested at (Florida Department of Agriculture and Consumer Services, 2025). The MODIS/Aqua Aerosol Cloud Water Vapor Ozone Monthly L3 Global 1Deg CMG (MYD08_M3) and the MODIS/Terra Aerosol Cloud Water Vapor Ozone Monthly L3 Global 1Deg CMG (MOD08_M3) data is from Platnick (2015). MERRA‐2 Hourly 0.625 × 0.5° V5.12.4 (M2T1NXFLX) data is from Global Modeling and Assimilation Office (GMAO) (2015). Automated Surface Observation Station data can be downloaded at: https://www.ncei.noaa.gov/products/land‐based‐station/automated‐surface‐weather‐observing‐systems. Meteorological data for the HYbrid Single‐Particle Lagrangian Integrated Trajectory (HYSPLIT) model was from the NOAA High‐Resolution Rapid Refresh (HRRR) model (Dowell et al., 2022). The HYSPLIT model can be run at: https://www.ready.noaa.gov/HYSPLIT.php. The interpolated monthly gridded output for the HYSPLIT model is also available (Sablan et al., 2025a).
