# Supplementary material for: Characterizing Particulate Matter Impacts of Smoke From 2022 to 2023 Agricultural Burning in South Florida
Source: Geohealth. 2026 Jan 27;10(1):e2025GH001365. doi: 10.1029/2025GH001365 (PMC12836378; doi:10.1029/2025GH001365)
Supplement: Supplementary file 1 — Supporting Information S1 [file GH2-10-e2025GH001365-s001.docx]

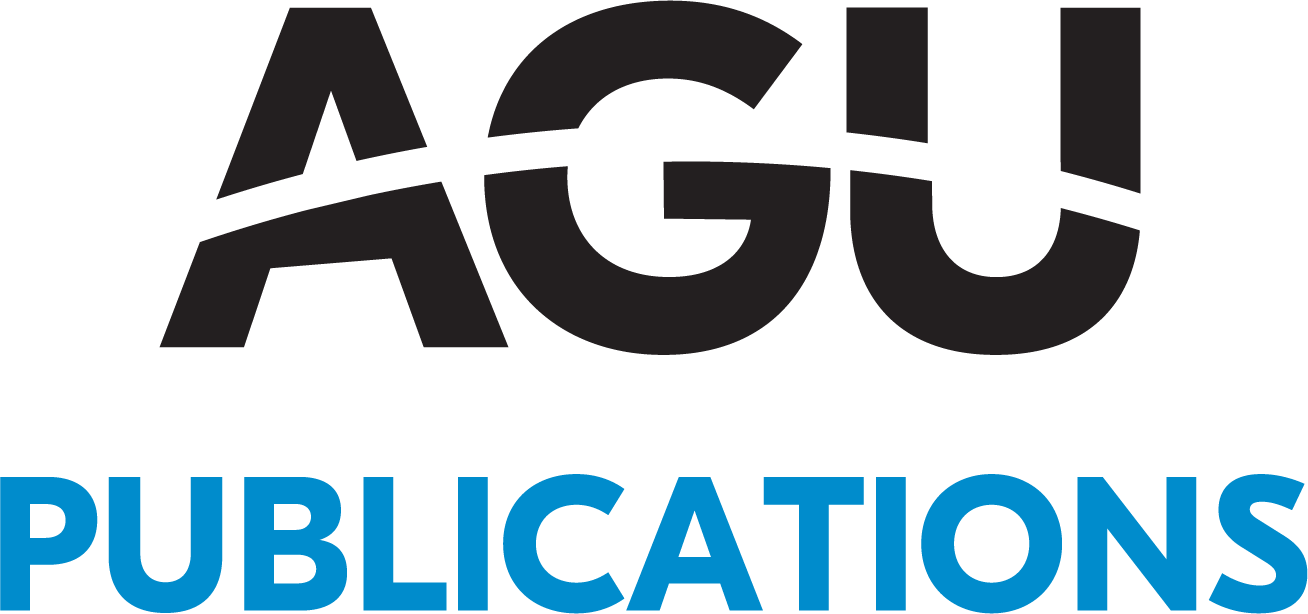


*AGU GeoHealth*

Supporting Information for

**Characterizing particulate matter impacts of smoke from 2022-2023 agricultural burning in south Florida**

Olivia Sablan^1^, Bonne Ford^2^, Emily Gargulinski^3,4^, Giovanna L. Henery^5^, Holly Nowell^6^, Zoey Rosen^7^, Kellin Slater^8^, Amber J. Soja^3,4^, Lisa K. Wiese^9^, Christine L. Williams^9^, Sheryl Magzamen^8^, Emily V. Fischer^1^, Jeffrey R. Pierce^1^

^1^Colorado State University, Department of Atmospheric Science

^2^ Colorado State University, Cooperative Institute for Research in the Atmosphere

^3^National Institute of Aerospace

^4^NASA Langley Research Center

^4^ Colorado State University, Department of Journalism and Media Communication

^5^ Tall Timbers

^7^ University of Oklahoma, Institute for Public Policy Research and Analysis

^8^Colorado State University, Department of Environmental and Radiological Health Sciences

^9^ Florida Atlantic University, College of Nursing

**Contents of this file**

Text S1 to S2

Figures S1 to S20

Table S1

**Introduction**

This supplemental material provides additional context and details essential for a comprehensive understanding of various aspects related to our findings. It details the pre-deployment quality checks of PurpleAir monitors, comparison of correction factors applied to PurpleAir field data, the timing, locations, and area burned for open burned authorizations, a specific smoke day example to further support our methods, cloud fraction from MODIS, figures from the main text plotted for other designation methods, diurnal of smoke and fires, tracking of HYSPLIT trajectory and monthly comparisons, and a comparison of PurpleAir and regulatory monitor PM_2.5_ measurements. Overall, this supplemental material aims to offer a transparent view of the research done in this project along with providing additional context.


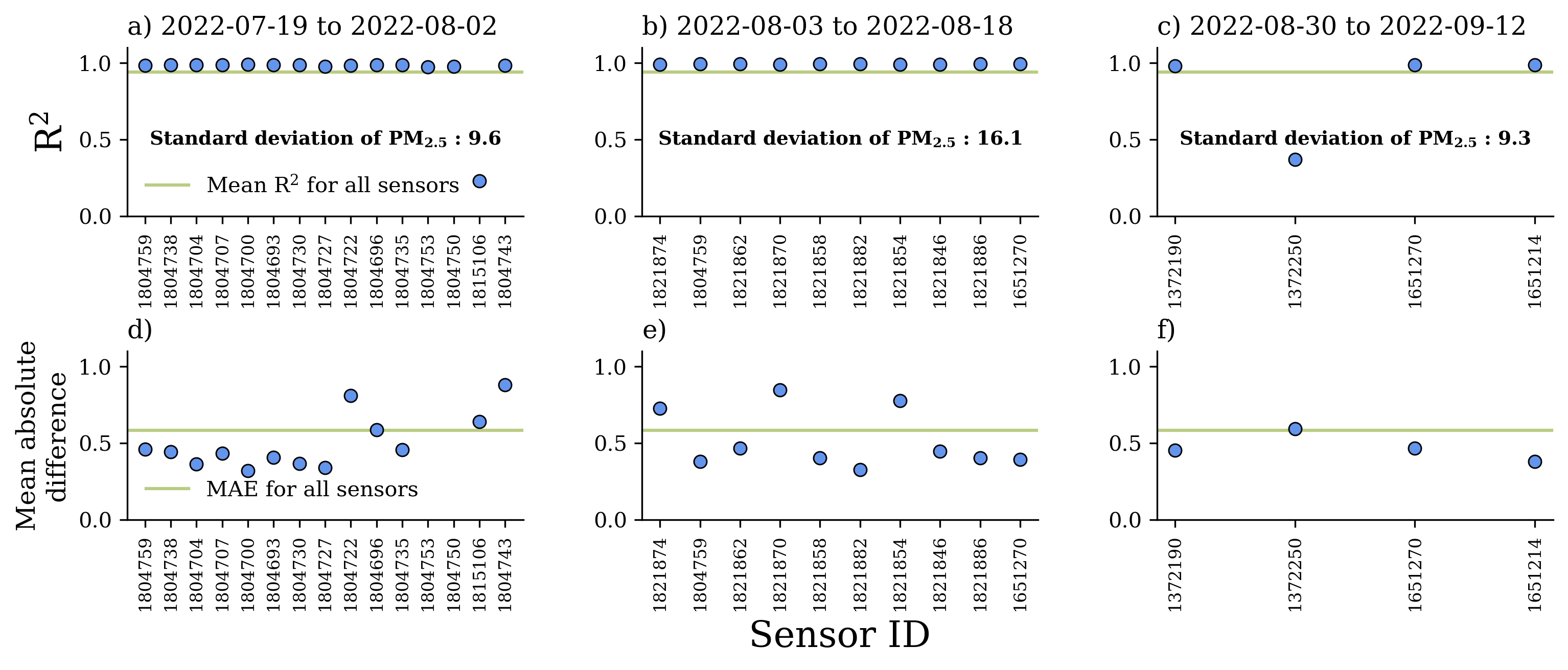


Figure S1. Pearson correlation between corrected PurpleAir channel A and channel B in comparison to the average correlation of all sensor channels (horizontal green line) for testing period 1 from 2022-07-19 to 2022-08-02 (a), testing period 2 from 2022-08-03 to 2022-08-18 (b), and testing period 3 from 2022-08-30 to 2022-09-12 (c). The mean absolute difference between channels is also provided for each testing period.

***
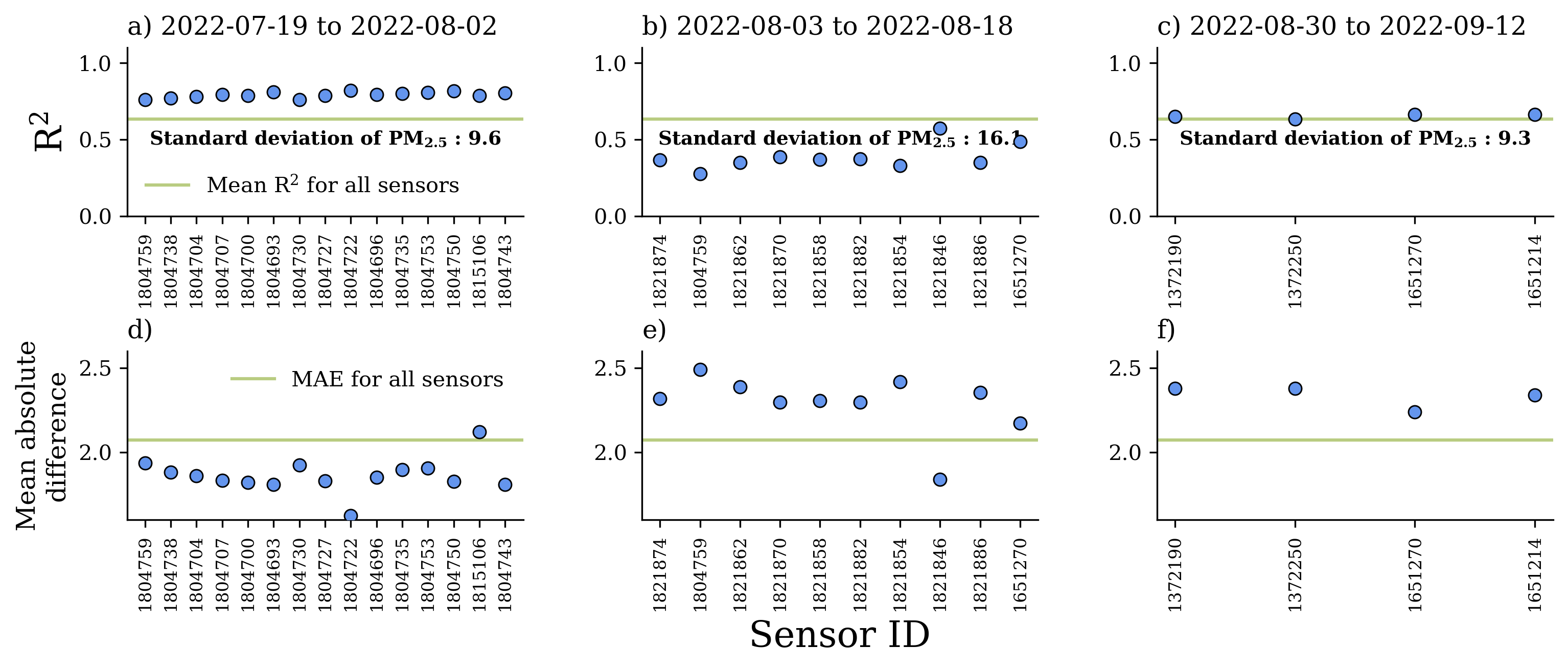
***

**Figure S2.** Pearson correlation between corrected 10-minute averages of PurpleAir sensors and the GRIMM monitor in comparison to the average correlation of all sensors (horizontal green line) and the GRIMM testing period 1 from 2022-07-19 to 2022-08-02 (a), testing period 2 from 2022-08-03 to 2022-08-18 (b), and testing period 3 from 2022-08-30 to 2022-09-12 (c). The mean absolute difference between channels is also provided for each testing period.


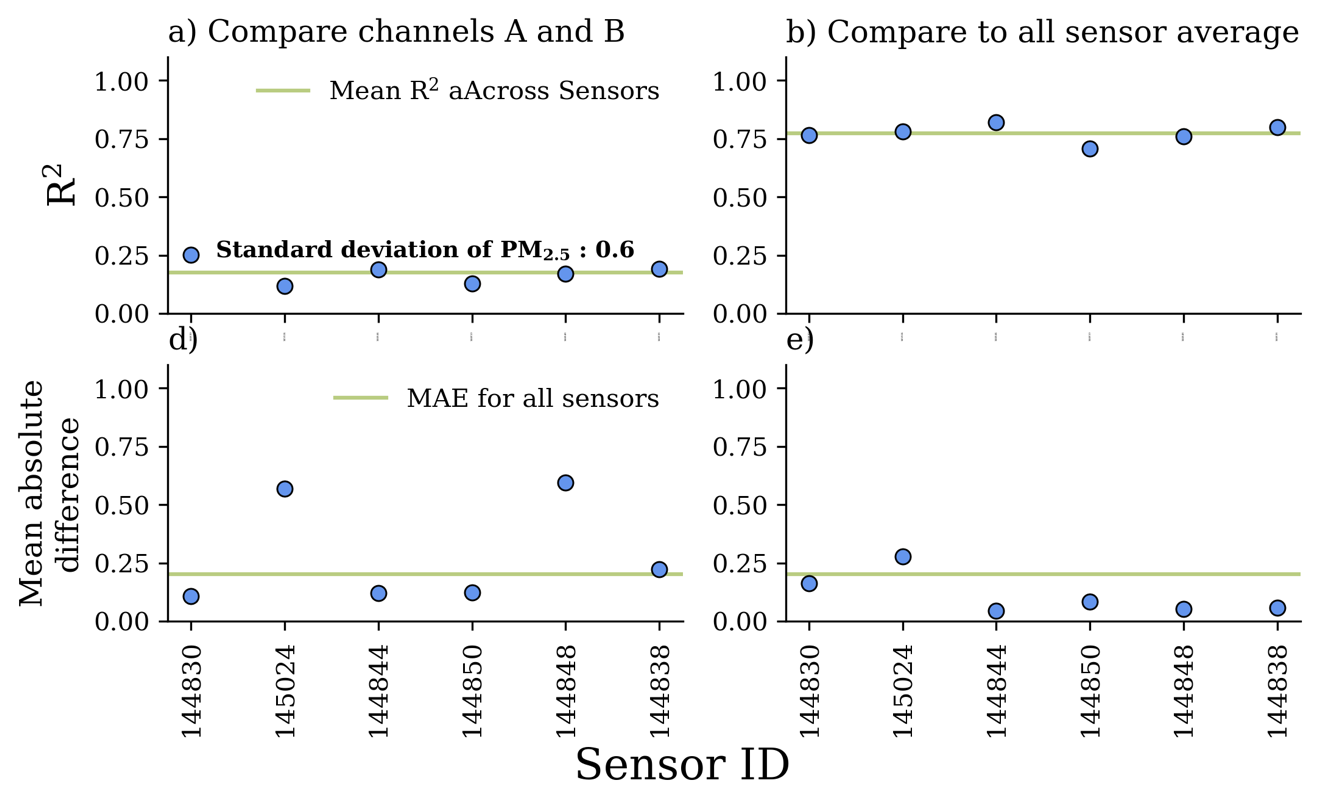


Figure S3.  Pearson correlation for brief testing (1 day) between 10-minute averages of the corrected PurpleAir sensor channels (a) and between the individual sensor channel average and the overall monitor average (b). The average correlation of all sensors for each test is represented by the green horizontal line. The mean absolute difference between channels (c) and the average (d) is also provided.


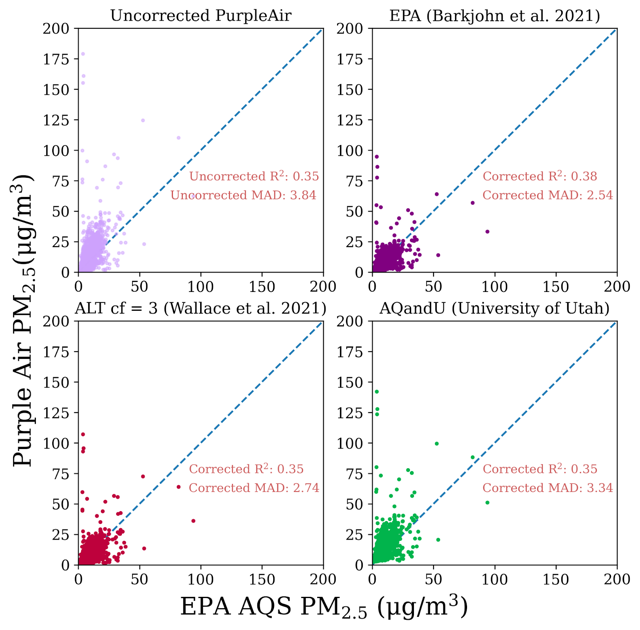


Figure S4.  Comparison of the hourly average of 14 Belle Glade PurpleAir monitors deployed by CSU and the EPA regulatory monitor in Belle Glade (AQS Site ID: 12-099-0008) with uncorrected PurpleAir data, Purple Air data with the Barkjohn et al. (2021) correction factor, the ALT [cf = 3] correction factor (Wallace et al. 2021), and the AQandU correction factor (Kelly et al., 2017; Sayahi et al., 2019). The Pearson correlation (R^2^) and mean absolute difference (MAD) is displayed in each figure for the corresponding data.


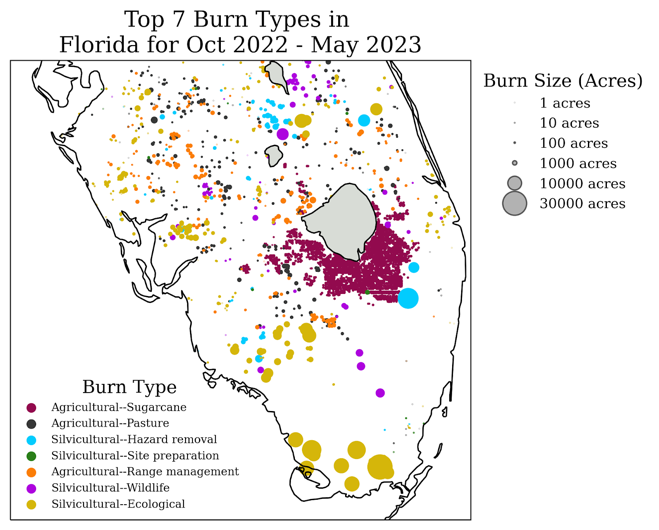


**Figure S5.** Map of study region including only the seven burn types with the most acreage burned from the Florida Fire Service Open Burn Authorizations. Markers are colored by burn type and sized by the acreage burned for October 2022 - May 2023.


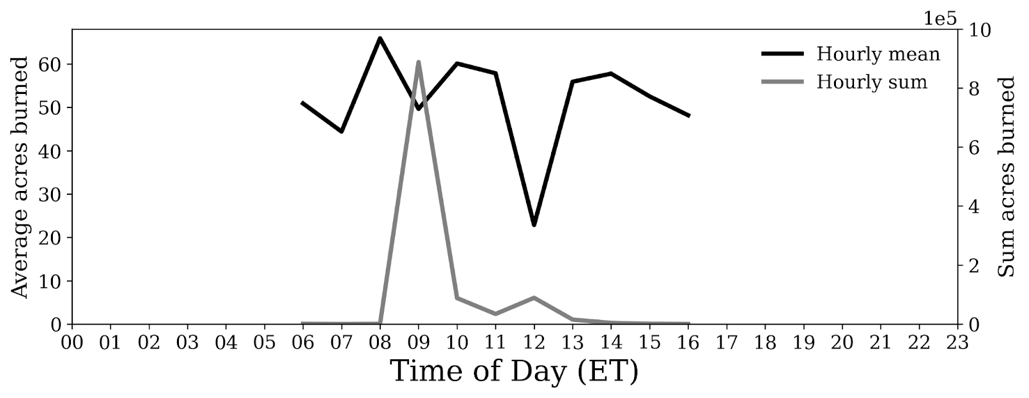


**Figure S6.** Hourly average acres burned (black) and hourly sum of acres burned (gray) from Florida Fire Service Open Burn Authorizations of sugarcane agricultural burning in the study region (≤ 27.5^o^) for October 2020 - May 2023.


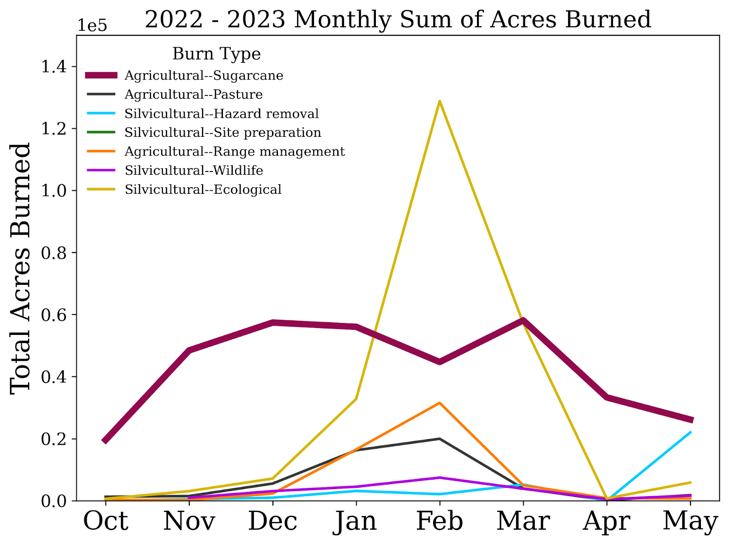


**Figure S7.** Monthly acres burned from sugarcane-agricultural fires from the Florida Fire Service Open Burned Authorizations for October 2022 - May 2023 in the study region (< 27.5^o^ N).

Text S1.

While other studies have relied solely on the HMS product to designate smoke, the limitations of HMS smoke plumes in the study region made it unreliable for consistently identifying smoke from agricultural fires in Florida. For example, on October 6, 2022, there were no HMS smoke plumes reported, although the increased PM_2.5_ concentrations in Belle Glade and HMS fire hotspots suggest the presence of smoke from agricultural fires (Figure S8). The average inland-monitor PM_2.5_ concentrations across all monitors increased from a minimum of 3.5 µg m^-3^ at 02:00 ET to 58.6 µg m^-3^ at 04:00. There was not a corresponding increase in PM_2.5_ measured at coastal monitors on this day, with the minimum average across all coastal monitors occurring at 00:00 (2.2 µg m^-3^) and the maximum occurring at 23:00 (5.2 µg m^-3^). There was some variability between monitors in each region, with a maximum PM_2.5_ for an individual inland monitor of 133.7 µg m^-3^ at 07:00 EST; however, the increase in PM_2.5_ overnight is also present in the inland monitor average. We have confidence there was smoke on October 6, 2022, due to the short-lived increase in PM_2.5_ observed with the inland monitors. The daily average of monitors in the city of Belle Glade, within the EAA, (inset in Figure S8) was 22.3 µg m^-3^ versus 5.9 µg m^-3^ for all other monitors on this day. Smoke plumes were not reported by HMS, likely because smoke occurred overnight when satellite observations were not available. If we relied on HMS smoke plumes to designate smoke impact, this day would be considered smoke-free.


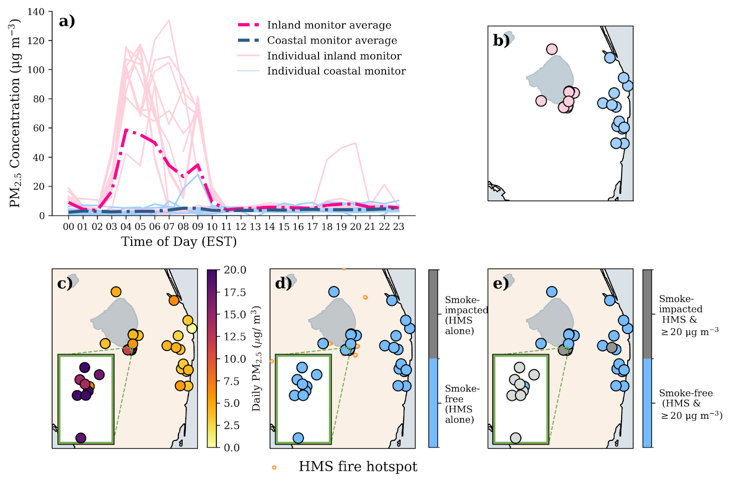


**Figure S8.** a) Hourly averages of individual inland monitors (light pink) and the average of all inland monitors (dark pink) on 6 October 2022 compared to hourly averages of individual coastal monitors (light blue) and the average of all coastal monitors (navy). We categorized monitors as coastal (<30 km from the coastline) or inland (>50 km from the coastline), with the inland monitors being closest to the EAA. b) Map showing the monitor separation (inland versus coastal), c) 24-hr average PM_2.5_ concentrations with an enlarged area of the monitors clustered in a small area (7.5 km^2^) near the city of Belle Glade, d) HMS fire hotspots are shown, while HMS smoke plumes are not available.  c) our own smoke designation as described in Section 2.4 of the main text.


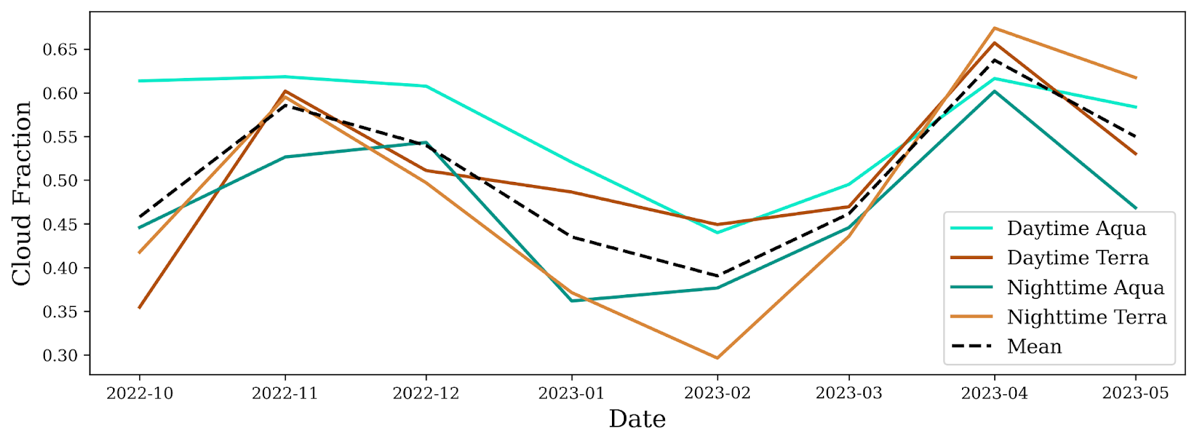


**Figure S9.** Time series from October 2022 - May 2023 of the cloud fraction from the day and nighttime MODIS product for Aqua and Terra. This is an area average of southeastern Florida which considers the region within longitudes 81°W to 80°W and latitudes 25°N to 27°N. The average of all 4 datasets is represented by the black dashed line.


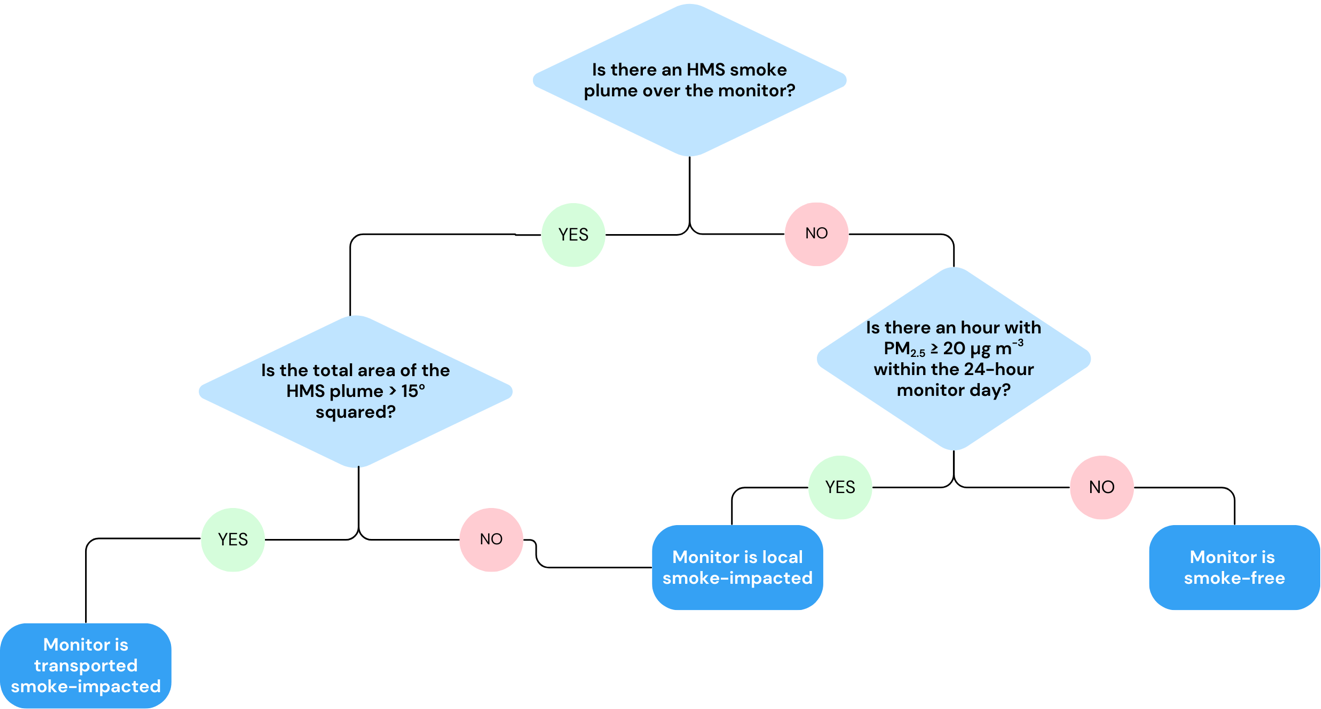


**Figure S10.** Flowchart detailing the “20 µg m^-3^ or HMS” smoke designation category.


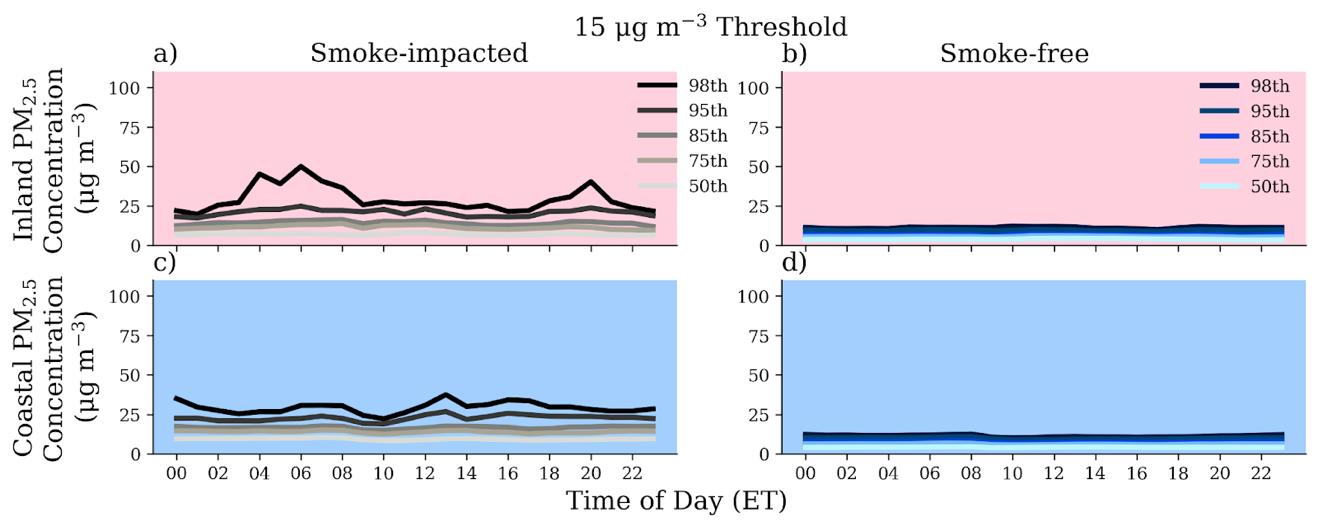


**Figure S11.** Hourly 50th, 75th, 85th, 95th, and 98th percentile PM_2.5_ concentrations during October 2022 - May 2023 for a) inland monitors (> 50 km from the coastline; pink) on smoke-impacted days (classified using criteria of an hourly average PM_2.5_ concentration ≥ 15 µg m^-3^), b) inland monitor on smoke-free days, c) coastal monitors (<30 km from coastline; blue) on smoke-impacted days, and d) inland monitors on smoke-free days.


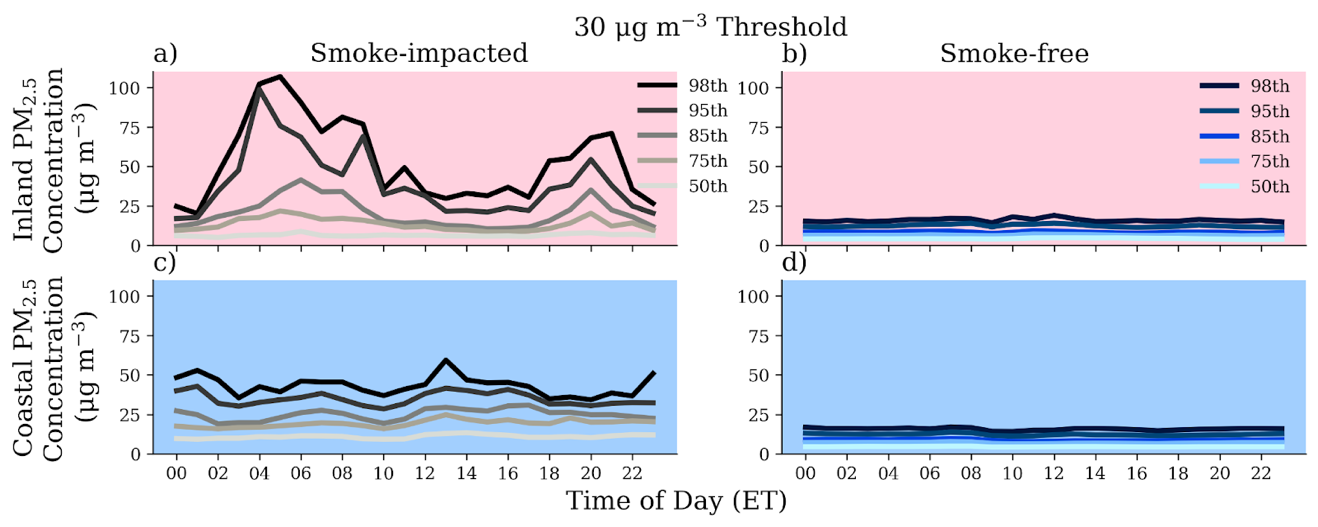


**Figure S12.** Hourly 50th, 75th, 85th, 95th, and 98th percentile PM_2.5_ concentrations during October 2022 - May 2023 for a) inland monitors (> 50 km from the coastline; pink) on smoke-impacted days (classified using criteria of an hourly average PM_2.5_ concentration ≥ 30 µg m^-3^), b) inland monitor on smoke-free days, c) coastal monitors (<30 km from coastline; blue) on smoke-impacted days, and d) inland monitors on smoke-free days.


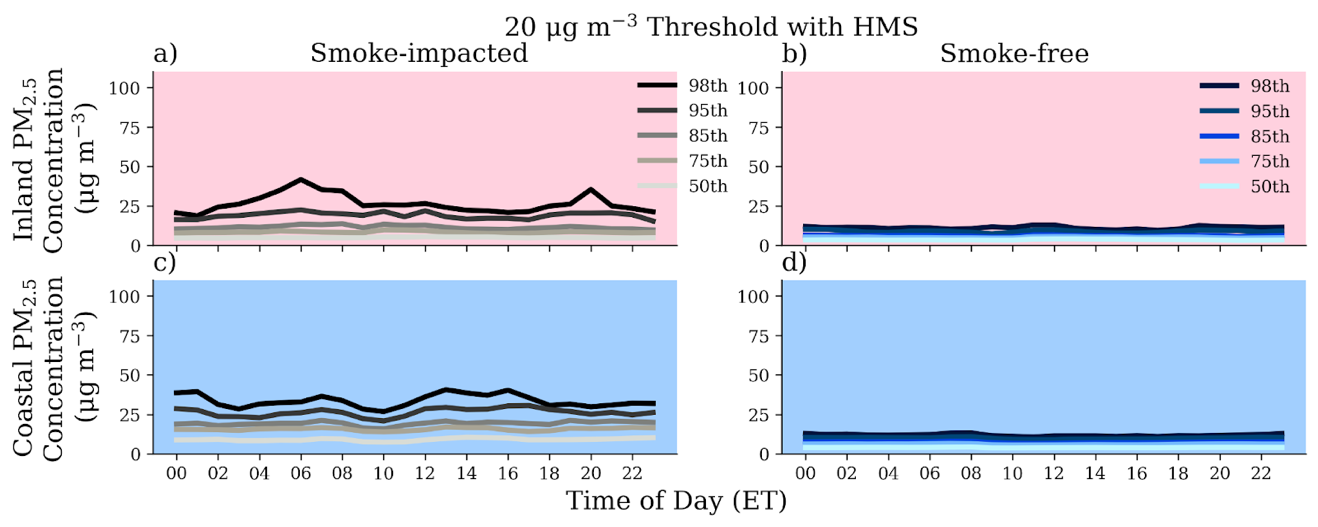


**Figure S13.** Hourly 50th, 75th, 85th, 95th, and 98th percentile PM_2.5_ concentrations during October 2022 - May 2023 for a) inland monitors (> 50 km from the coastline; pink) on smoke-impacted days (classified using criteria of an hourly average PM_2.5_ concentration ≥ 20 µg m^-3^ and HMS), b) inland monitor on smoke-free days, c) coastal monitors (<30 km from coastline; blue) on smoke-impacted days, and d) inland monitors on smoke-free days.


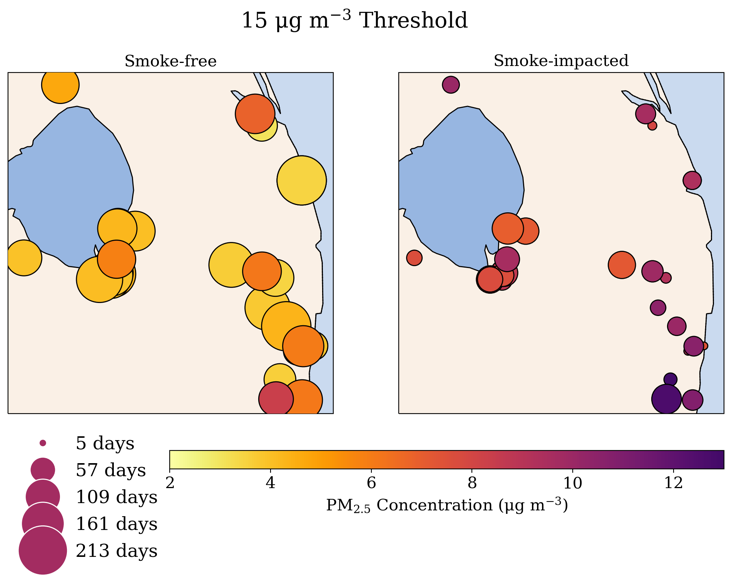


**Figure S14.** Maps of median PM_2.5_ concentrations at each monitor for the ≥ 15 µg m^-3^ designation method on smoke-free (left) and smoke-impacted days (right). Markers are sized by the corresponding number of monitor days. Map regions are shaded by inland versus coastal. Note: the color bar is initiated at 2 µg m^-3^.


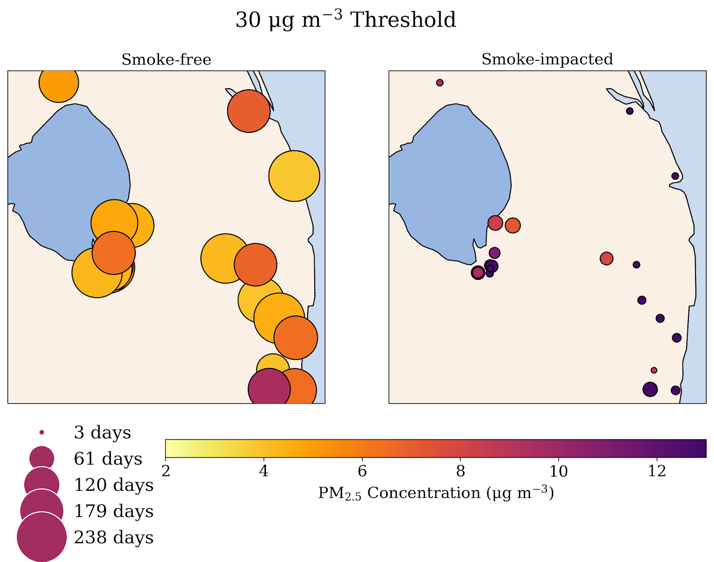


**Figure S15.** Maps of median PM_2.5_ concentrations at each monitor for the ≥ 30 µg m^-3^ designation method on smoke-free (left) and smoke-impacted days (right). Markers are sized by the corresponding number of monitor days. Map regions are shaded by inland versus coastal. Note: the color bar is initiated at 2 µg m^-3^.


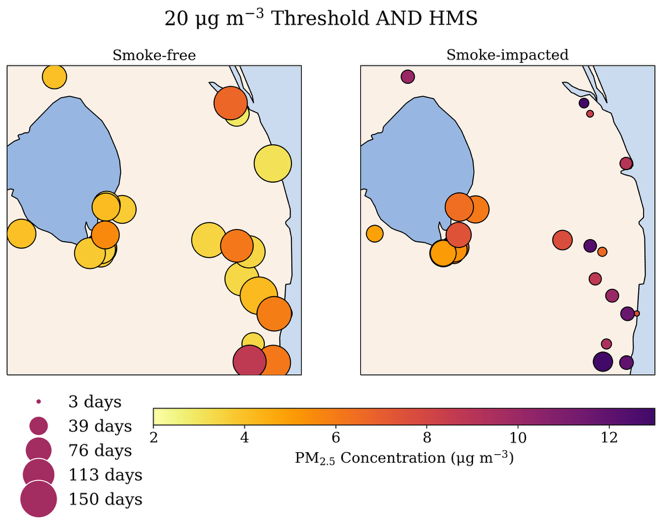


**Figure S16.** Maps of median PM_2.5_ concentrations at each monitor for the ≥ 20 µg m^-3^ and HMS designation method on smoke-free (left) and smoke-impacted days (right). Markers are sized by the corresponding number of monitor days. Map regions are shaded by inland versus coastal. Note: the color bar is initiated at 2 µg m^-3^.


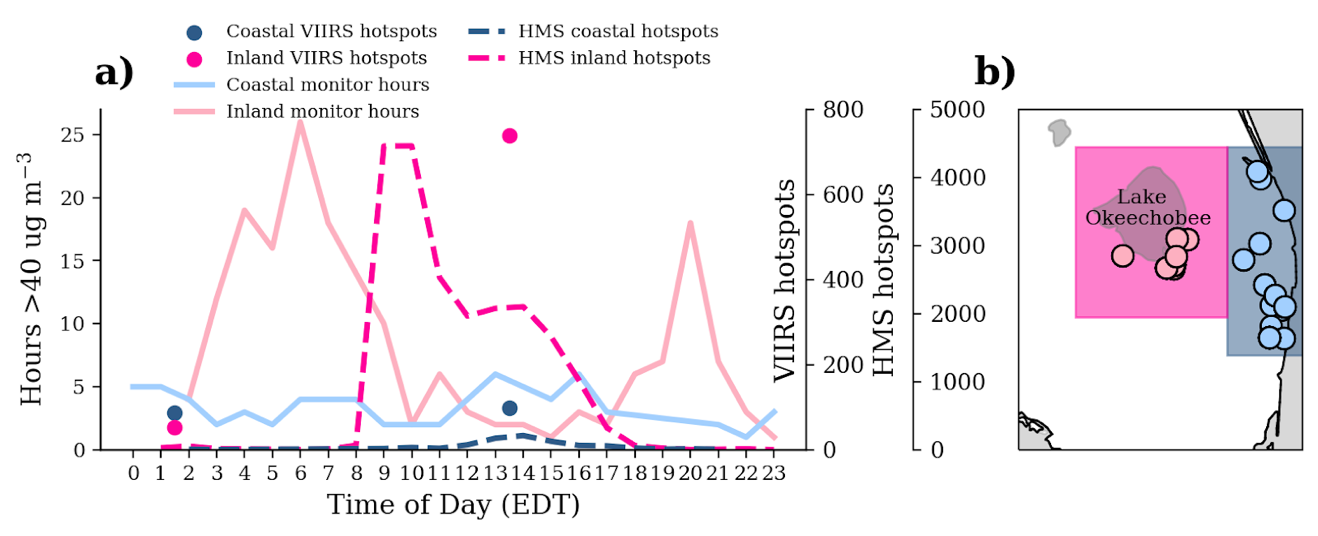


**Figure S17.** Hourly averages of the number of HMS hotspots detected in the inland region and within the pink shaded region on the map (dark pink), and hotspots detected near the coast and within the blue shaded region on the map (navy). Hourly averages of the count of monitors that exceed 40 µg m^-3^ in the inland region (light pink) and near the coast (light blue). The monitor locations are colored by region on the map.


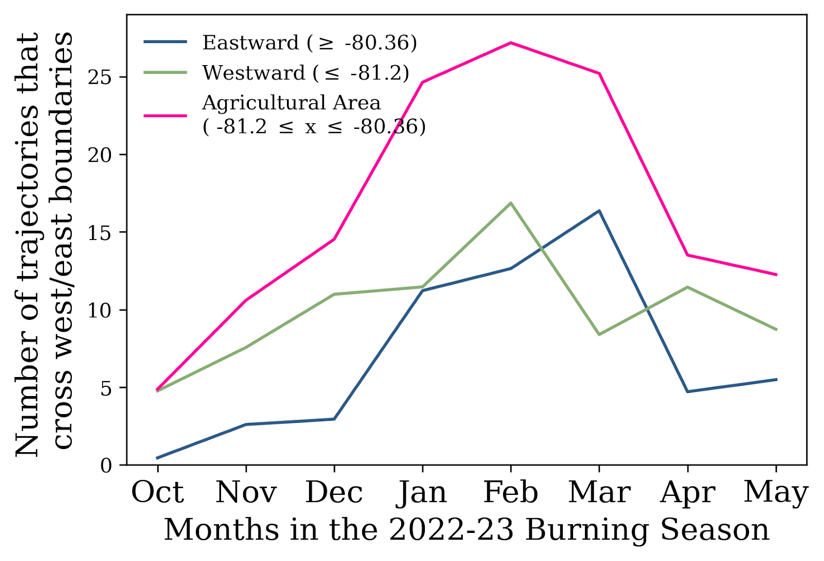


**Figure S18.** Number of HYSPLIT trajectories initiated from every HMS hotspot in southern Florida that moves eastward, with a longitude greater than or equal to -80.36^o^ W (blue), that moves westward, with a longitude less than or equal to -81.2^o^ W (green), or that stays in the agricultural area close to the EAA (-81.2^o^ ≤ x ≤ -80.36^o^W) (pink) for October- May of 2022-23. Trajectories that cross the boundaries multiple times are only counted once.

**Text S2.**

From October-December, February, and April-May, more HYSPLIT trajectories moved westward (Figure S18). During March, when there was the greatest number of monthly detected hotspots (25), more trajectories moved eastward than westward (2106 versus 902). This suggests that in most months, smoke from agricultural burning primarily impacts regions to the west, potentially reducing smoke exposure along Florida’s eastern coastline. However, the significant eastward transport during March, shows variability and complexity in smoke transport.


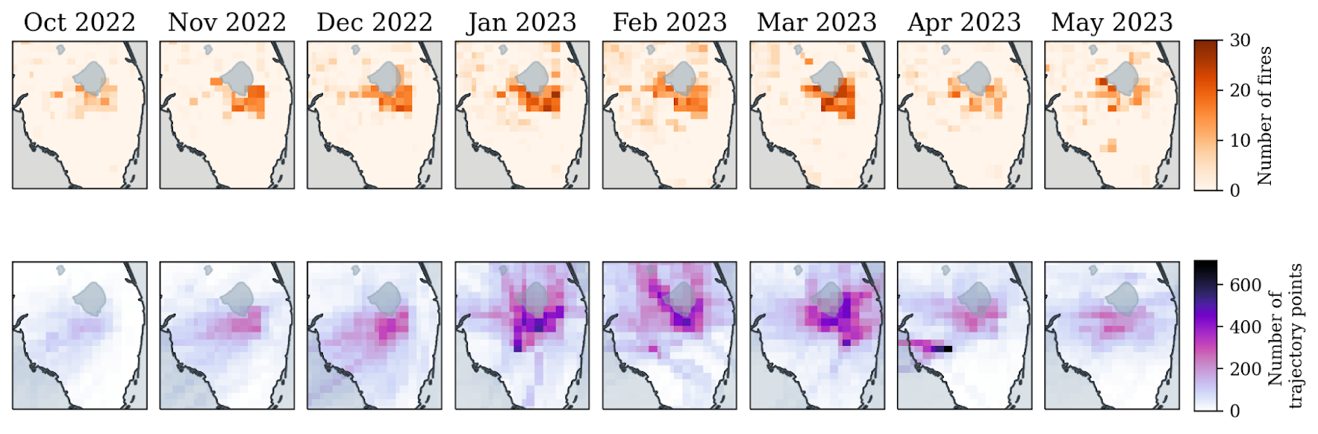


**Figure S19.** Gridded HMS fire hotspots for the 2022-23 burning season and 10-min interpolated 12-hour forward trajectories run from each HMS fire hotspot from the NOAA HYSPLIT model.


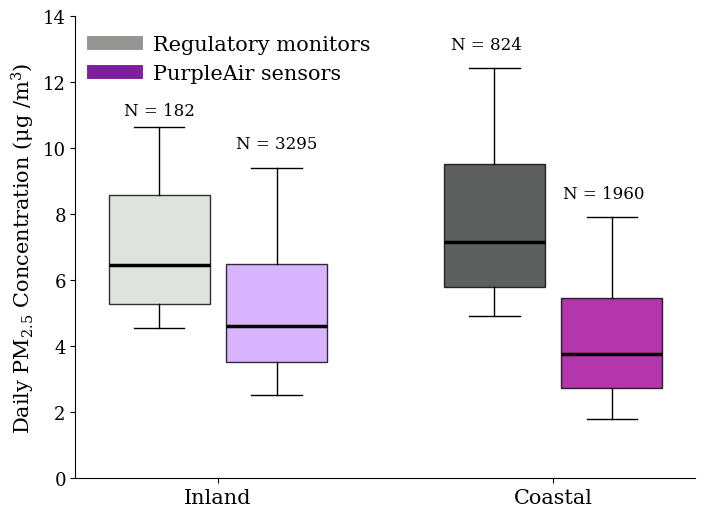


**Figure S20.** Daily average PM_2.5_ for inland monitors versus coastal monitors days. The distributions are separated by regulatory monitors from the EPA AQS (grays) and PurpleAir sensors from the study deployment and public sensors (purples). Outliers have been excluded and the bold line represents the median for each category. The edges of the box represent the 1st and 3rd quartiles. The whiskers are 10th and 90th percentiles.
